# Multiscale Characterization and Bioactivity of Freshwater Unionid Mussel Shells as Sustainable Natural Biomaterials

**DOI:** 10.1021/acsomega.6c02594

**Published:** 2026-06-01

**Authors:** Kerim Emre Öksüz, Hülya Şereflişan, Erkan Uğurlu

**Affiliations:** † Department of Metallurgical and Materials Engineering, Sivas Cumhuriyet University, Sivas 58140, Turkey; ‡ Institute of Science and Technology, Department of Bioengineering, Sivas Cumhuriyet University, Sivas 58140, Turkey; § Faculty of Marine Sciences and Technology, 450201 İskenderun Technical University, İskenderun 31200, Hatay, Turkey

## Abstract

The complex biological material of the freshwater mussel’s shell serves various functions, contributing to the support of ecosystem services. Beyond offering structural integrity and reinforcement, the shell emerges as a significant natural biomaterial candidate thanks to its distinctive composition and structure. The aim of this study was to investigate the potential use of freshwater mussel shells from two unionid species *Anodonta anatina* (Linnaeus, 1758) and *Unio delicatus* (Lea, 1863) as a natural biomaterial. The shells were examined for their chemical composition, physical characteristics, and mechanical strength, in addition to undergoing a thorough analysis of their biointerface structure and surface morphology. The major mineral composition of CaCO_3_ and the presence of other elements in their ionic forms in both mussel species demonstrated a favorable association with the shell’s composition, microstructure, biointerface, biomineralization, and biological responses. The mineralogical/biointerfacial changes observed in various parts of the mussels were clearly depicted in the FE-SEM micrographs. Additionally, studies involving XRD, XRF, FTIR, EDXS, and Raman spectroscopy were used to confirm the presence of CaCO_3_ crystals (aragonite and calcite forms) in the mussel shells along with the detection of organic components with various functional groups. Furthermore, in vitro bioactivity and hemocompatibility assays were conducted to investigate the effect of shell composition on the biological properties of samples of freshwater mussel shells. The results demonstrated that shell samples with different contents facilitated fibroblast attachment, migration, and promoted cell proliferation. These findings also verified the hemocompatibility of the shell samples and the favorable impact on the hemostatic behavior of the shells mineralogical structure. The findings indicated that the inherent shells of freshwater mussels *Anodonta anatina* and *Unio delicatus* are distinctive biological materials that hold promise for various applications benefiting human well-being and contributing to the preservation of environmental quality.

## Graphical abstract

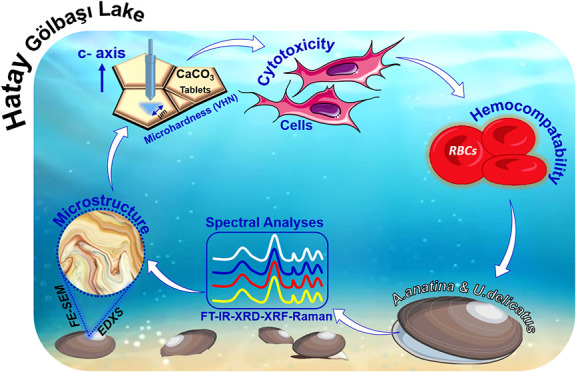

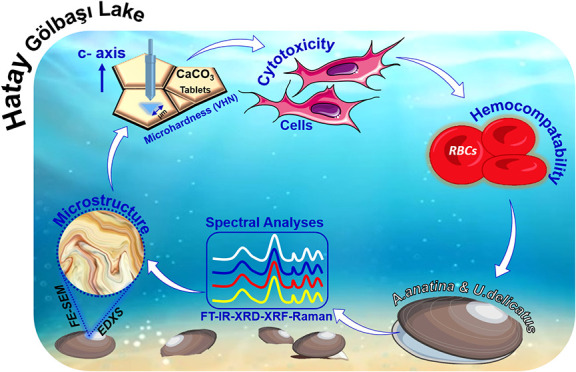

## Introduction

Unionid freshwater mussels, classified in the Mollusca phylum, hold significant importance within freshwater ecosystems. They are recognized as beneficial creatures, primarily due to the services they provide to the ecosystem and their exceptional functions.^1^ Furthermore, mussels possess biomonitoring features that offer evidence concerning the current and historical hydrological conditions of aquatic ecosystems.^2^ Unionid mussels, distinguished by their unique life cycle,^3^ rely on host fish during their parasitic stage.^4,5^ As a result, the presence of fish populations is also vital for ensuring the sustainability of mussels in the freshwater ecosystem. Nevertheless, a robust global protection strategy is imperative for safeguarding these mussels against life-threatening factors, whether environmental or human-induced.^6^ Changing environmental factors have a significant impact on both the life cycle and phenotypic variability of the shell structure in freshwater mussels.^7^ The biological activity of a shell is influenced by its chemical composition and structural organization, which determine its strength, durability, and protective functions. Mechanical properties like hardness, elasticity, and surface texture affect their interaction with the environment and resistance to physical stresses. Additionally, the shell’s ability to grow, repair itself, and its biochemical composition play vital roles in maintaining integrity and interacting with the organism’s tissues. Mussels, which possess a biomineral exoskeleton, exhibit a unique hierarchical structure that enhances their mechanical resistance to adverse environmental conditions.^8^ The mantle predominantly forms the shell of the mussel, which is composed of at least 95% CaCO_3_ in the form of calcite and/or aragonite.^9^ Furthermore, due to their high CaCO_3_ content and natural hierarchical architecture, mussel shells have emerged as sustainable precursors for advanced biomaterial applications. These naturally occurring materials are being used as osteoconductive scaffolds for bone tissue engineering, bioactive fillers in dental composites, and as a calcium source for hydroxyapatite synthesis, facilitating enhanced integration with living tissues. The morphology, structure, and various adaptations of mussel shells play a crucial role in determining their capacity to flourish in an ever-changing environment. Shell characteristics have historically played a central role in the comprehensive study and description of freshwater mussels. Researchers in Türkiye, Syria, and the Middle East have used these distinctive features to conduct various studies on the demographic and biogeographical distribution of species.^6^ However, in recent times, there has been an increasing acknowledgment that depending solely on the morphology of the shell for systematic descriptions of mussels may lead to potential misinterpretations. Currently, the modern taxonomy of freshwater mussels adopts an integrative approach that incorporates not only conchological features but also other characteristics, such as molecular and anatomical data.^10^ The matter of diversity, distribution, and evolutionary development of Unionid mussels holds particular significance in the Western Palearctic region, as well as on a global scale. Although the documentation of species diversity and distribution is comprehensive in northern, western, and central Europe within this geographical area, substantial knowledge gaps persist, particularly in the Balkans, Türkiye, and the Middle East.^11^ Efforts to bridge this information gap have led to intensive Unionid mussel diagnosis in the Eastern Mediterranean, including Türkiye and neighboring countries. As part of these efforts, the distribution of *U. delicatus* (Lea, 1863) and *A. anatina* (Linnaeus, 1758) (Bivalvia; Unionidae) in Türkiye, Israel, and Iran was determined through phylogenetic analysis and comprehensive taxonomic evaluation.^6^ The periostracum color of *A. anatina* varies, typically appearing brownish yellow, with white nacre, and inhabiting mud and clay substrata rich in organic matter. *U. delicatus*, on the other hand, exhibits a brownish outer shell layer with a brightly colored nacre on the inner shell layer and prefers muddy sediment as its habitat.

The aim of this experimental study is to conduct a thorough analysis of the morphological diversity exhibited by the microstructures and biological properties (in vitro) inherent to the shells of two distinct unionid species, *A. anatina* and *U. delicatus*. These particular species have been deliberately chosen from the vast array of freshwater mussels that populate Gölbaşı Lake, located in a picturesque region of Türkiye. Through this focused investigation, we aim to focus on the intricacies of their shell characteristics, unveil the remarkable array of microstructural patterns, and delve into the in vitro biological properties that make these species unique within this specific ecological context.

## Materials and Methods

### Study Area

The freshwater mussels *A. anatina* and *U. delicatus* were collected from Gölbaşı (Balık Gölü) Lake in the Kırıkhan district of Hatay, Türkiye, in July 2023. Gölbaşı Lake is located approximately 3–4 km from the Syrian border. Situated at the foot of the Kurt Mountains in the northeast of Amik Lake, it is 29 km from Reyhanlı and 11 km from Kırıkhan. Gölbaşı Lake, the only natural lake in Hatay, is within the borders of the Kırıkhan district, between Gölbaşı Village and Kamberlikaya Village. Additionally, there is a small natural island within Gölbaşı Lake. The wetland area of Gölbaşı Lake (36°30′17″ N–36°29′41″ E) is constantly supported by an underground water sources and covers approximately 325 ha. Fishing is conducted in the sampled lake. Gölbaşı Lake is rich in biodiversity and is classified as a mesotrophic lake according to the Brachionus/Trichocerca index. It is recognized as an important habitat for *A. anatina* and *U. delicatus*.^12^ Figure 1 presents the location and coordinate details of the sample collection area.

**Figure 1:**
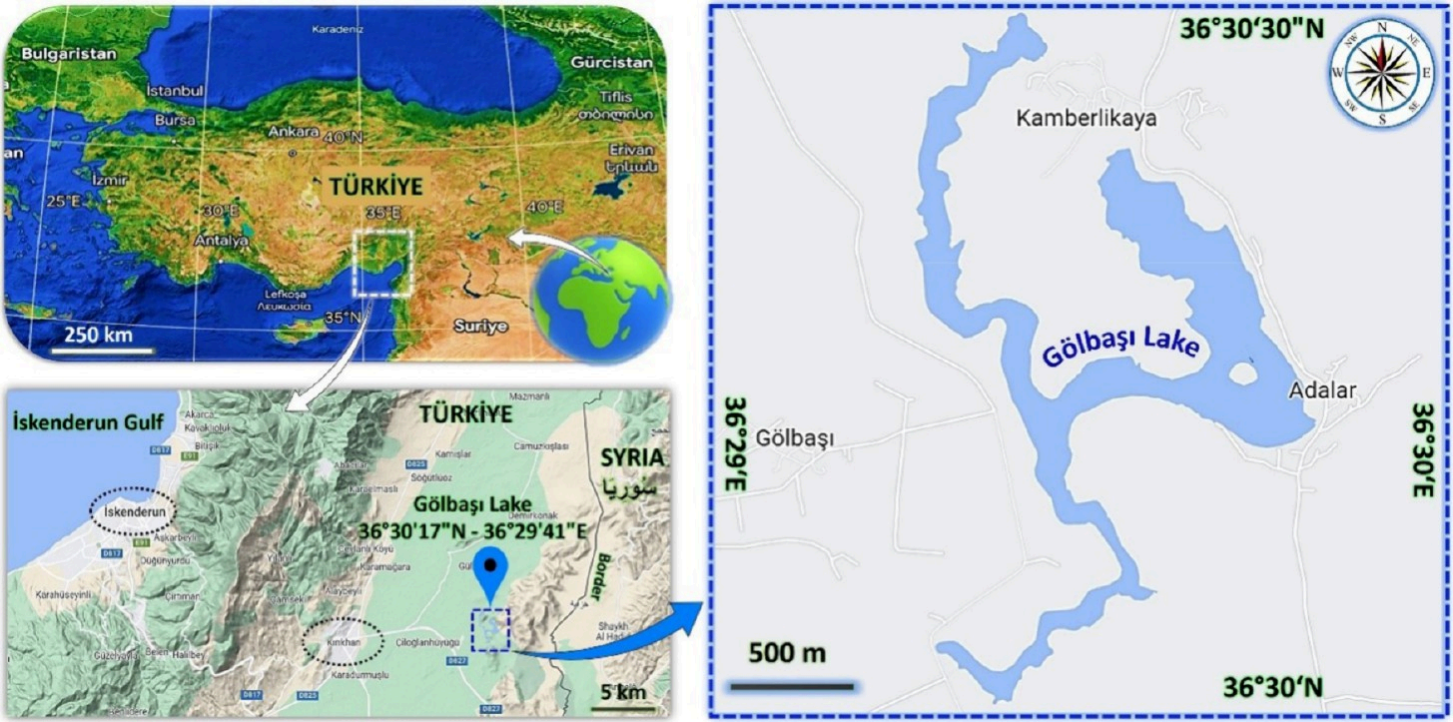
Geographical location and detailed mapping of Gölbaşı Lake (Hatay, Kırıkhan, Türkiye), where freshwater mussel shells are collected.

### Preparation of Shell Samples

Following sample collection and identification, the soft tissues were meticulously excised using a scalpel. The remaining shells underwent a thorough cleaning process under running water, were disinfected with ethanol (C_2_H_6_O), and were subsequently left to sun-dry for 3 days. Additionally, any organic substances clinging to the shells were carefully removed to avoid adversely affecting the experimental results.^13,14^ Subsequently, the shell length and height of the samples were measured using a digital caliper with a precision of 0.05 mm. A summary of the shell characteristics of the studied species is given in Table 1.^6^

**Table 1:** Distribution of *A. anatina* and *U. delicatus* Freshwater Mussel Shells by Country

species	*n*	length (cm)	height (cm)	distribution
*A. anatina*	20	10.74 ± 0.52	3.77 ± 0.22	Türkiye, Azerbaijan, Armenia, Iran, Syria
*U. delicatus*	20	7.50 ± 0.49	2.91 ± 0.25	Türkiye, Lebanon, Syria

### Characterization of Freshwater Mussel Shells

#### Fourier-Transform Infrared Spectroscopy (FT-IR) Analysis

FT-IR spectroscopy (Bruker Alpha II FT-IR Spectrometer, Germany) was used to characterize the surface chemistry of freshwater shell samples. For both *A. anatina* and *U. delicatus*, 100 mg of dried mussel shell powder was mixed separately with 1% KBr. The mussel shell powder was ground to produce KBr pellets for FT-IR analysis. The analyzed spectral range scanned from 400 to 4000 cm^–1^, with a wavenumber resolution of 4 cm^–1^. Infrared radiation was employed to identify and record the chemical bond vibrations of the shell samples; subsequently, data acquisition and processing were performed using the OPUS spectroscopic software.

#### X-ray Fluorescence Analysis (XRF)

XRF spectroscopy (Thermo Scientific, Niton, XL3t, U.S.A.) was employed to carry out both qualitative and quantitative analyses of the inorganic compositions of calcined freshwater mussel shell powders, with a focus on the major oxides. In order to determine the amount of oxide present in mussel shells, the shell powder was calcined at 900 °C for 3 h at a heating–cooling rate of 5 °C/min, consistent with the procedure reported by Gao et al.^15^

#### X-ray Diffraction Analysis (XRD)

The crystalline phase, constituting the major component of the shell powder from freshwater mussels *A. anatina* and *U. delicatus*, was identified through powder XRD analysis. A Rigaku D/MAX/2200/PC diffractometer (Rigaku, Austin, USA), was used to collect data. Monochromatic Cu–Kα radiation (λ = 1.5408 Å) was employed, and the scan range was from 20° to 80° with a step size of 0.02. and a scan rate of 1°/min. This approach was employed to characterize the crystal structure of the mussel shell powders.^16,17^ The different phases were determined from ICDD (International Centre for Diffraction Data) database using MDI JADE software.

#### Raman Spectroscopy of Freshwater Mussel Shells

The Renishaw inVia Raman Microscope was used to record Raman spectra from 400 to 4000 cm^–1^.^18^ The measurement configuration followed a backscattering arrangement. Excitation was carried out using a Renishaw 532 nm helium–neon laser with a maximum output power of 17 mW. Data acquisition was performed using an advanced CCD array comprising 1024 × 256 pixels. To ensure optimal performance, the CCD array was cooled by a Peltier device and kept at −70 °C. To eliminate elastically scattered laser light, a notch filter was added. The laser power was carefully controlled at 0.085 mW/μm^2^ over a 20 μm^2^ area. Stringent precautions were taken to safeguard the sample and prevent any laser-induced damage. In all spectral analyses, prepared dried mussel shells were randomly selected in two pairs. After grinding the shells with a mortar and pestle, they were passed through a 45 μm sieve. Spectral analyses were then performed on the samples obtained in homogeneous powder form.

#### Vickers Microhardness (VHN) Testing

Micromechanical tests were carried out in accordance with ASTM C1327–08 standards using Shimadzu HMV-2000-model 3212 VHN hardness tester under a load of 0.1 kg-f and a dwell time of 15 s. This test was carried out using two different samples per species in both prismatic and nacreous layers for the microhardness measurements of the shells. To evaluate the Vickers microhardness of mussel shells, selected shells were cut using a diamond disc and cooling water in a precision cutting machine (Brilliant 220-ATM GmbH). The cutting conditions were constant at *n* = 3500 rpm and *fz* = 3 mm/min. The cut shells were sliced in a horizontal position and the specimens were embedded with epoxy to ensure homogeneity in the loading direction for microhardness testing. The epoxy resin was removed from the shell specimen surface by grinding. After grinding, it was carefully polished with abrasive papers and lubricants. The embedding and polishing of the mussel shell segments were performed using standard metallographic techniques as described in the referenced literature.^19^ This technique facilitated separate tests within both prismatic and nacreous layers. For each sample, 12 microhardness values were taken in both layers, the average values were calculated and statistical analysis was performed. In the hardness measurements, the load applied on the shells was equally spaced across the width of the shell segment in the prismatic and nacreous layers. Each experiment was performed for in at least two repetitions and VHNs were automatically calculated and expressed by the micromechanical testing machine.^20,21^

#### Composition and Microstructural Aspects of Mussel Shells (FE-SEM, EDXS)

For detailed examination of surface morphology and interfacial properties, *A. anatina* and *U. delicatus* mussel shells were broken into separate pieces and the samples with the flattest surface were selected. The broken pieces were subjected to a cold mounting process in order to detail the interfacial properties. The resin-embedded shell samples were then removed from the cold mounting mold, and the resin blocks were subjected to a rigorous grinding and polishing process with aluminum oxide (Al_2_O_3_) sandpaper ranging from 320# to 2000#. They were super mirror polished with a metallographic grinding automatic polishing machine (Qpol 250 M1/2, Germany) using Al_2_O_3_ (≥5 μm) and polycrystalline diamond paste (3, 1, and 0.5 μm). To reach the successive inner layers of the mussel shells, ethylene diaminetetraacetic acid (EDTA), a weak chemical etching solution, was applied to the surfaces of the shell fragments for 3 min at RT. Subsequently, the samples were decontaminated in an ultrasonic bath using ethanol for 15 min at 30 °C and then dried on both surfaces with a blow dryer.^22^ Field Emission Scanning Electron Microscopy (FE-SEM, Tescan Mira3 XMU, Brno, Czech Republic) was utilized to examine *A. anatina* and *U. delicatus* freshwater mussel shell samples, layer thickness, surface morphology, homogeneity and interface structure. Each shell sample was coated with a gold–palladium alloy for 10 min at 20 mA with 15–20 kV. Determination of the elemental composition of the shell samples was carried out using an Energy dispersive spectroscopy instrument coupled to FE-SEM (Oxford Inca Energy 350 EDXS, UK).

#### In Vitro Biocompatibility Studies

Cytotoxicity of shell samples of *A. anatina* and *U. delicatus* was assessed using standard ISO 10993–5 test procedures. XTT reagent (2,3-bis­(2-methoxy-4-nitro-5-sulfophenyl)-2H-tetrazolium-5-carboxanilide) colorimetric assay was used to evaluate the antiproliferative activity of samples. L929 mouse fibroblast cell lines (commercial standard cell line originated from mouse fibroblast cells, ATCC # CCL-1, Thermo Fisher Scientific, USA) were used for cytotoxicity assays. Cells were cultured in Dulbecco’s Modified Eagle Medium (DMEM) supplemented with a preprepared solution in a protected medium. These cells were incubated in sterile conditions for 24 h and then seeded in a 96-well microplate at a density of 1 × 10^4^ cells/well and incubated again at 37 °C for 48 h (at 37 °C, 90% humidity, 5% CO_2_ atmosphere). Freshwater mussel shell powders were sterilized for 24 h before XTT testing. The cells underwent treatment with different concentrations (2, 4, 6, and 10 μL) and were subsequently incubated in a 37 °C environment with 5% CO_2_ for a duration of 48 h. Following incubation, the culture medium was extracted, and the wells were washed three times with phosphate-buffered saline (PBS). Subsequently, 200 μL of DMEM, along with 50 μL of a 5 mg/mL XTT reagent, was introduced into each well and left to incubate for an additional four hours. Absorbance values of the cell solution were measured at a wavelength of 450 nm using a Thermo Scientific Multiskan FC Microplate Photometer reader (USA). The experiments were performed in triplicate.^23–26^

#### Hemocompatibility Assay

To evaluate the hemocompatibility of freshwater mussel shells (*A. anatina* and *U. delicatus*), we determined the hemolytic activity values (%) based on literature studies.^27,28^ For the hemocompatibility assay, 10 mL of fresh sheep blood was obtained from a slaughterhouse, and EDTA (1.5 mg/mL, ethylenediaminetetraacetic acid) was added to prevent coagulation. Subsequently, the fresh blood was centrifuged at 5000 rpm for 3 min at 4 °C. The resulting healthy red blood cells (HRBCs) were washed in PBS with a pH of 7.4 and diluted 10-fold with a sterile PBS solution. Following this, 2 mL of the resuspended HRBCs in PBS (pH 7.4) and 25 mg of sterile mussel shell powder were added to each blood tube. The blood mixtures were incubated for two hours at a temperature of 37 °C with gentle shaking under sterile conditions. Afterward, the blood tubes were centrifuged at 2000 rpm for 10 min, and the absorbance of the supernatants corresponding to hemoglobin was measured at 541 nm using an ultrafast UV/vis spectrometer (BMG LABTECH, Spectro Star Nano, Germany). Positive and negative controls were prepared for all hemolytic activity assays. The positive control consisted of 1.6 mL of sterile dH_2_O mixed with 0.4 mL of HRBCs, while the negative control consisted of 1.6 mL of sterile PBS mixed with 0.4 mL of HRBCs. For the hemolytic activity values, each sample was verified at least twice and the average values of the experiments are presented. The following equation was used to calculate the hemolytic activity (HA):^1^

$$\mathrm{HA}=\frac{{\mathrm{OD}}_{s}-{\mathrm{OD}}_{\mathrm{NC}}}{{\mathrm{OD}}_{\mathrm{PC}}-{\mathrm{OD}}_{\mathrm{NC}}}\times 100\%$$ 1

where OD_S_, OD_NC_, and OD_PC_ denote the absorbances of the tested sample, the negative control sample, and the positive control sample, respectively.

#### Blood Clotting Assay

The study employed a modified version of a previously reported method^27,29^ to evaluate the blood coagulation efficacy of *A. anatina* and *U. delicatus* mussel shells, quantified as the blood clotting index (BCI). The procedure for this process was as follows: Fresh blood from healthy sheep was obtained at a slaughterhouse using a blood tube containing an anticoagulant and promptly transported to the biomedical laboratory. Samples of mussel shell powder were placed in the center of glass Petri dishes. Then, fresh sheep blood (100 μL) was gently deposited on its surface. Immediately afterward, 10 μL of a 0.2 M CaCl_2_ solution was promptly added, acting as a catalyst for clot formation. The samples were subsequently placed in a warm 37 °C incubator for 20 min, enabling the blood to initiate its clotting process. After the incubation, 5 mL of sterile dH_2_O was carefully added to each dish, avoiding disturbance of the newly formed blood clot. The mixture of blood and dH_2_O was collected from the plates using a pipet and transferred to polypropylene test tubes. The centrifuged supernatants were transferred to sterile tubes. The tubes were incubated for one hour at 37 °C. At the end of the incubation period, the optical density of the samples was measured at 540 nm using an ultrafast UV/vis spectrometer (BMG LABTECH, Spectro Star Nano, Germany). Sterile distilled dH_2_O was used as the base for optical density measurements. A blend of 5 mL of sterile dH_2_O and 100 μL of blood served as a positive control. The blood coagulation capability of each sample was validated in duplicate, and the average of two separate experiments was employed to determine the blood clotting index (BCI) according to eq 2:^2^

$$\mathrm{BCI}=\frac{{\mathrm{OD}}_{s}}{{\mathrm{OD}}_{\mathrm{Pc}}}\times 100\%$$ 2

where OD_S_ and OD_PC_ denote the absorbances of the tested sample and the positive control sample, respectively.

#### Statistical Analyses

The collected data are presented as means ± standard deviations (SD) based on at least three (*n* = 3) independent measurements. All data were statistically analyzed using one-way ANOVA, followed by the Dunn’s or Dunnett’s posthoc tests where appropriate. Statistical significance was determined at *p ≤ 0.05 and **p < 0.001 using the OriginPro 9.0 software package.

## Results and Discussion

### FT-IR Spectroscopy Analysis

In Figure 2, the FT-IR spectra of the raw powders of *A. anatina* and *U. delicatus* shell samples are displayed. The characteristic OH—stretching peak at 3675 cm^–1^ is prominent and sharp in *U. delicatus*, whereas it appears at a lower intensity in the shell samples of *A. anatina* and is not clearly detected. Additionally, both mussel shell samples exhibit a C–H stretching band in the 2897 and 2370 cm^–1^ regions.^30^ The sharp peak observed at 713 cm^–1^ and the weak minor band detected at 1797 cm^–1^ are identified as the combination bands of the *v*_4_ + *v*_1_ vibrational modes.^31^ These spectral features provide strong evidence for the presence of organic components (amides) in the sample. The sharp peaks at 713 and 691 cm^–1^, in particular, are indicative of specific vibrational transitions associated with amide functional groups, reinforcing the conclusion drawn from the spectral analysis.

**Figure 2:**
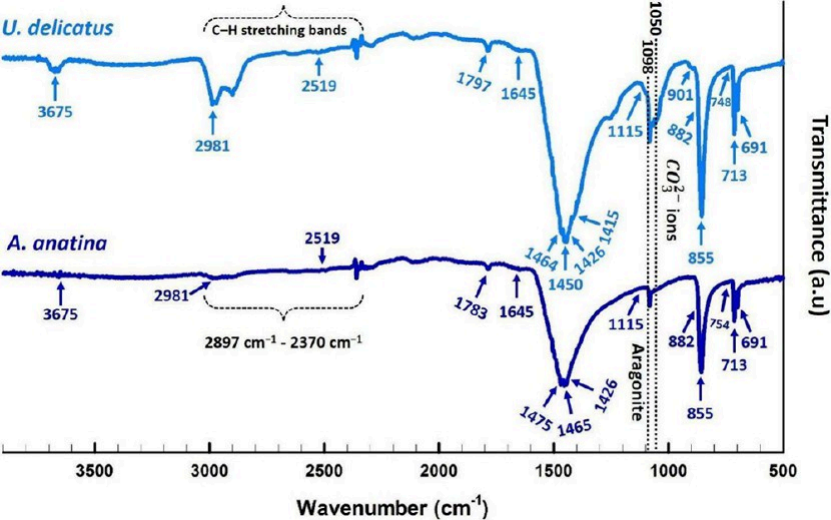
FT-IR spectra for the surfaces of *A. anatina* and *U. delicatus* freshwater mussel shell samples. Through the analysis of infrared absorption patterns, the spectra offer a comprehensive examination of the functional groups existing on the surface of the mussel shells, revealing information about the organic and inorganic components that contribute to their physicochemical properties.

A sharp peak at 855 cm^–1^ and a doublet at 1465/1450 cm^–1^ unequivocally identify *A. anatina* and *U. delicatus* mussel shell samples, respectively, as containing carbonates (CO_3_^2–^). The vibrational frequencies for CO_3_ at 855 cm^–1^ for *v*_2_ and 1645, 1465, and 1415 cm^–1^ for *v*_3_ provide clear evidence of their presence. Two types of carbonate were clearly observed: A-type, resulting from substitution between CO_3_ and the OH—site, with characteristic peaks centered at 1540 cm^–1^;^32^ and B-type, resulting from substitution at the PO_4_ site, with corresponding regions around 855, 1415, 1465, and 1450 cm^–1^.^33^ The band at 855 cm^–1^ confirms carbonate substitution for the PO_4_^3–^ group (B-type substitution),^33,34^ indicating the presence of B-type carbonate in both freshwater mussel shell samples. Deviations in the *v*_3_ carbonate bands do not affect this conclusion. The absorption peak associated with the *v*_1_ symmetric stretching mode of the CO_3_^2–^ carbonate ion was observed in both samples, specifically at 1050 and 1098 cm^–1^ within the crystalline aragonite structure.^35^

Besides, the *v*_4_ IR band is particularly useful for identifying (CO_3_^2–^) minerals. This band distinctly shifts in position depending on the cation type, with the peak occurring at 713 cm^–1^ for CaCO_3_ (calcite) and at 748/754 cm^–1^ for MgCO_3_ (magnesite).^36,37^ The band position for intermediate compositions typically lies between the corresponding bands for calcite and magnesite. Isomorphic substitutions cause only a slight shift in the carbonate absorption bands. Mg is typically derived from both seawater and freshwater sources. High-Mg-calcite is an unstable mineral (CO_3_^2–^) phase compared to low-Mg-calcite. Over time, it may lose its Mg content and transition into low-Mg-calcite. According to the literature, peaks observed at 2981, 1797, 1415, and 863 cm^–1^ are characteristic of low-Mg-calcite, while the peak at 2519 cm^–1^ is indicative of high-Mg-calcite.^38^ In the FT-IR analyses, it was observed that the low-Mg-calcite peaks in the *U. delicatus* mussel shell samples appear more sharply and distinctly than those in *A. anatina* mussel shells.

The IR absorption bands, including three split bands around 855 and 882 cm^–1^ neighboring bands around 1415 cm^–1^ (more prominent in *U. delicatus* samples) and 1475 cm^–1^, as well as a single band around 1115 cm^–1^, can be attributed to Mg carbonates.^39^ The bands at 1450 and 901 cm^–1^ (indicating C=O bond stretching), along with a single band at 855 cm^–1^ and a band at 1098 cm^–1^, suggest the presence of (CO_3_^2–^) from SrCO_3_, while characteristic carbonate peaks at 1426, 882, and 713 cm^–1^ indicate the presence of Na_2_CO_3_ minerals.^40^

### X-ray Fluorescence Analysis (XRF)

XRF measurements were conducted to qualitatively determine the elemental compositions of natural *A. anatina* and *U. delicatus* shell samples. The results indicate that both shell powders are primarily composed of CaO.^15^ This highlights the potential of shells as a valuable biosource of highly pure CaCO_3_. As indicated in Table 2, the CaO content in *U. delicatus* shell powders was notably high at 97.48 wt %, while XRF analysis identified other oxides in *U. delicatus* shells, including MgO, K_2_O and SiO_2_.^41^ Other oxides, such as MnO, CuO, Fe_2_O_3_ and P_2_O_5_, were present at levels below 0.1 wt %. In contrast, *A. anatina* shell powders exhibited a CaO content of 95.59 wt %, along with relatively higher levels of other oxides, such as MgO, K_2_O, SiO_2_, MnO and lower levels (≤0.1 wt %) of oxides like CuO, Fe_2_O_3_, and P_2_O_5_ were detected compared to *U. delicatus* shell powders. Based on the XRF data, it is evident that mussel shell biofiller is predominantly composed of CaCO_3_, rendering it appropriate for various applications.

**Table 2:** XRF Results of the *A. anatina* and *U. delicatus* Shell Samples (wt %: Weight Percent)

freshwater mussels	CaO (wt %)	MgO (wt %)	K_2_O (wt %)	SiO_2_ (wt %)	MnO (wt %)	CuO (wt %)	Fe_2_O_3_ (wt %)	P_2_O_5_ (wt %)
*A. anatina*	95.59 ± 0.105	3.761 ± 0.782	0.152 ± 0.029	0.136 ± 0.015	0.145 ± 0.002	0.068 ± 0.0021	0.087 ± 0.009	0.061 ± 0.002
*U. delicatus*	97.48 ± 0.215	2.031 ± 0.851	0.160 ± 0.03	0.154 ± 0.053	0.042 ± 0.0018	0.070 ± 0.003	0.040 ± 0.003	0.022 ± 0.002

### Phase Evolution by XRD Analyses

To gain insights into the structural/phase composition of mussel shells, XRD experiments were conducted. The XRD results of *A. anatina* and *U. delicatus* freshwater mussel shells are depicted in Figure 3. Comparative analysis of XRD patterns from shells of these distinct mussels uncovered resemblances in crystalline peaks (Figure 3), providing confirmation of the presence of both aragonite and calcite forms of CaCO_3_. It distinctly shows that the main crystalline phases of the both mussel shell samples are aragonite-(CaCO_3_) (JCPDS no. 76-0606) and minor calcite-(CaCO_3_) (JCPDS no. 86-2339) are detected for *U. delicatus* freshwater mussel shell powders, shown in Figure 3. The XRD pattern of *A. anatina* mussel shell owns more peaks with higher intensity than *U. delicatus* freshwater mussel shell powders. The XRD phase analysis of aragonite and calcite is depicted in Figure 3, showcasing intense peaks at 2θ = 26.11°, 27.11°, 31.01°, 33.01°, 36.00°, 37.18°, 37.78°, 38.49°, 41.08°, 42.81°, 48.31°, 50.12°, 52.32° and 52.89° along with low-intensity peaks at 2θ = 22.33°, 29.48° and 60.21° respectively with monochromatic Cu Kα radiation (λ = 1.5406 Å). In accordance with the definition of the prismatic and nacreous structure, the XRD results additionally affirm the distinctive microstructural characteristic of both natural mussel shells.^41,42^

**Figure 3:**
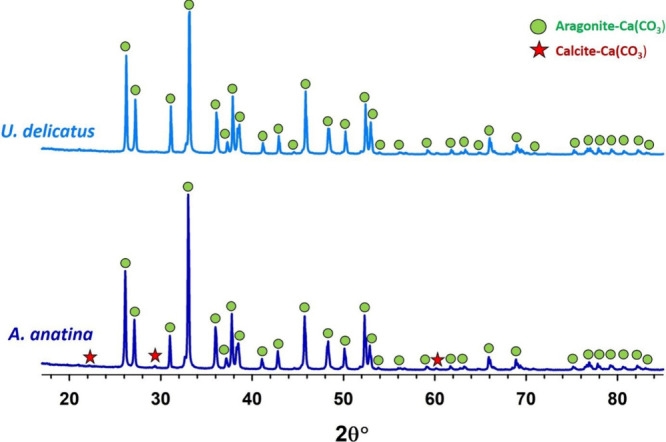
XRD diagrams of the *A. anatina* and *U. delicatus* freshwater mussel shell powders. For both mussel shell samples, prominent aragonite-(CaCO_3_) and calcite-(CaCO_3_) phases are observed.

### Raman Spectroscopy Analysis

Micro-Raman is primarily employed for studying the biomineralization of the structural composition of organic, inorganic, and biological molecules^43^ within the analyzed samples. Raman spectroscopy is an effective method employed to examine molecular vibrations, the biochemical composition and the biostructure of a material. The assignment of a particular band requires knowledge of the specific molecules or functional groups present in the sample and their characteristic vibrational frequencies. Typically, Raman spectroscopy analyses have been conducted on the powdered freshwater mussel shells. The powdered samples from *A. anatina* and *U. delicatus*, representing the freshwater mussel shells, showed a variety of peak heights, confirming the presence of calcite and aragonite crystals, which is also supported by the Raman spectrum in Figure 4.

**Figure 4:**
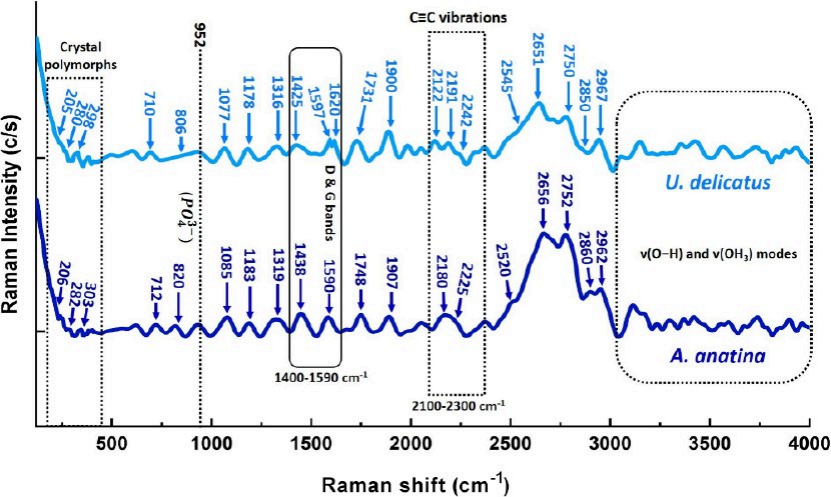
Raman spectroscopy analysis of *A. anatina* and *U. delicatus* freshwater shell samples revealing the presence of two types of aragonite and calcite crystals in the both specimens. The bands labeled as D and G correspond to the rectangular line. The spectra of both samples exhibit shared bands at 952 cm^–1^ which correspond to PO_4_^3–^ groups. The main bands in the range of 2100–2300 cm^–1^ are combined with the C≡C (triple bond stretch) vibrations in both samples.

The band positions of all the Raman modes of calcite found in mussel shell samples precisely match the values of calcite reference bands.^44^ Typically, calcite crystals contain two CaCO_3_ units, corresponding to a total of ten atoms. Their nucleation and crystal growth behaviors can be thoroughly analyzed using Raman spectroscopy and theoretical calculations.^45^ In the 200–305 cm^–1^ region of the Raman spectrum used for detecting crystal polymorphs, regarding the mineral composing both mussel shells, all the Raman spectra confirm the presence of calcite with characteristic bands at around 280–305 cm^–1^ (CO_3_^2–^ groups), 710–712 cm^–1^ (*v*_4_, asymmetric bending), and 1077–1085 cm^–1^ (*v*_1_, symmetric stretching of CO_3_^2–^ groups) as the main band.

For the *A. anatina* and *U. delicatus* samples, which are predominantly composed of calcite/aragonite the bands around 1425/1438 and 1731/1748 cm^–1^ ^52^ are observed as good agreement with Gaspard et al. in the literature^46^ (Figure 4). Conversely, the Raman spectrum for aragonite shows similar vibration modes to those of calcite, but with a slight shift to lower energies for all modes. The crystal contains four CaCO_3_ units, for a total of 20 atoms; the presence of the Raman-active modes at 205–206, 701–705, 1085, 1178–1183, and 1316–1319 cm^–1^ has been reported for aragonite crystalline powder for both mussel shell powders.^47,48^ In the literature, Dufresne et al.^49^ examined eight different Raman spectra of calcite structures and compared their data with previously reported results. They obtained results similar to ours for the Raman spectra of calcite structures synthesized by the hydrothermal method and those found in natural sources. Urmos et al.^50^ studied biogenic carbonates from a scleractinian coral (*Porites* sp.), pink-pigmented (*Corallium regale*), and unpigmented (*Corallium secundum*) precious corals, as well as natural and cultured pearls. They reported that the Raman spectra of aragonitic coral and pearls exhibited bands specific to aragonite, with shifts and changes in these bands suggesting either small crystallite size or crystal structure disorder. In another detailed study, Wehrmeister et al.^51^ analyzed vaterite in freshwater cultured pearls using Raman spectroscopy. Their findings were consistent with ours, as they successfully identified polymorphs of CaCO_3_ in the Raman spectra, distinguishing between aragonite, calcite, and vaterite.

The Raman spectra of calcium phosphate (Ca_3_(PO_4_)_2_) minerals are dominated by a strong band especially for *A. anatina* mussel shell samples at around 960 cm^–1^ that derives from the symmetric stretching mode (*v*_1_) of the phosphate (PO_4_^3–^) group. This corresponds to the *v*_1_(P–O) vibration of the PO_4_^3–^ group within a crystalline network.^46^ Sauer et al.^52^ characterized the organic and mineral components of biological and synthetic Ca_3_(PO_4_)_2_ minerals using Raman spectroscopy and they reported that the position of this band varies with different mineral standards. It is detected at 952 cm^–1^ for amorphous calcium phosphate, 957 cm^–1^ for octacalcium phosphate, 960 cm^–1^ for hydroxyapatite, and 985 cm^–1^ for dicalcium phosphate dihydrate. Additionally, they noted that the band at 952 cm^–1^ for noncrystalline amorphous calcium phosphate is three to four times wider than that observed for crystalline calcium phosphates. In the Raman spectra shown in Figure 4, it can be observed that the bandwidth corresponding to *U. delicatus* sample is broader than that of the *A. anatina* sample.

Both mussel shell samples, which exhibit partially black shells, contain various pigments. The Raman spectrum obtained resembles that of melanin, closely aligning with the spectrum of amorphous carbon. It also exhibits the characteristic broad D and G bands (Figure 4), observed around 1400 and 1590 cm^–1^. These bands are attributed to in-plane stretches of the aromatic rings and linear stretches of the C–C bonds linkages.^53^ In Figure 4, the bands at 1900 and 1907 cm^–1^ can be assigned to the symmetric stretching mode *v*_1_ + *v*_4_ (in-plane bending mode) in the *A. anatina* and *U. delicatus* freshwater shell samples respectively. Zakaria et al.^54^ conducted a study using FT-Raman and FT-IR spectroscopic methods on seashells of the *Philippine Venus* sea shell and sea coral of the *Porites* sp. both of which contain aragonite-structured CaCO_3_. Their findings were consistent with the results of our study.

The vibrations associated with C≡C (triple bond stretch) are observed within the spectral range of 2100–2300 cm^–1^ in the Raman spectra. Figure 4 also reveals the presence of two distinct Raman active peaks at 2520 and 2545 cm^–1^ in the SH stretching modes of mussel shells. SH···O and SH···S vibrations are responsible for these active Raman peaks.^55^ The difference in signal intensity in the high wavenumber region between *A. anatina* and *U. delicatus* sample is observed in the 2600 to 2700 cm^–1^ band attributed to the second vibrational mode (2D band).^56^ We observed high- frequency carbon–hydrogen (C**—**H) vibrations in the Raman spectrum of the *A. anatina* sample at 2656 cm^–1^ and in the *U. delicatus* sample at 2651 cm^–1^ in this study (Figure 5a).

**Figure 5:**
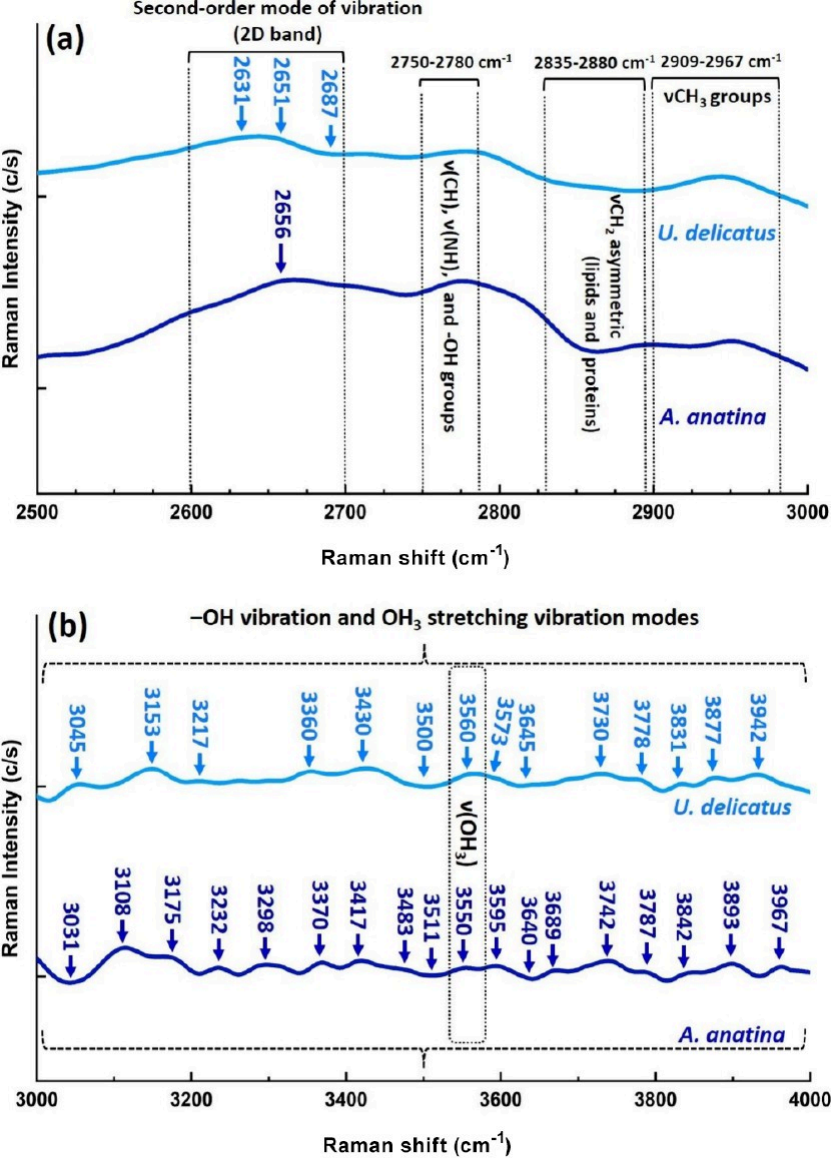
Raman band assignment in the (a) 2500–3000 and (b) 3000–4000 cm^–1^ spectral range for *A. anatina* and *U. delicatus* freshwater mussel shell samples. For both samples, prominent bands are observed. These bands can be attributed to specific molecular vibrations associated with the presence of certain functional groups or crystal structures like 2D bands, and *v*(CH), *v*(NH), *v*(CH_3_), *v*_as_(CH)_2_, *v*(OH^–^ *)* and *v*(OH)_3_ groups.

In their study, Bazylewski et al.^57^ employed Raman spectroscopy to differentiate between thiol modifications in l-cysteine, specifically targeting sulfhydryl’s in proteins and enzymes. They found that at a 1:1 ratio, *S*-methylmethanethiosulfonate completely converted all cysteine thiols into the product S1 (cystine) with disulfide bonds (they identified that above 2500 cm^–1^ in Raman as a CH and SH stretching modes). This conversion was evidenced by an inverse relationship between the intensities of the SH and SS stretching modes. Another study conducted by Dovbeshko et al.^58^ investigated Coherent Anti-Stokes Raman Scattering (CARS) of various carbon nanostructures, as well as CARS spectra of thymine molecules adsorbed on graphene oxide. The CARS spectra were compared with the spontaneous Raman scattering spectra of these samples, revealing that the spectral bands observed for the complex materials exhibited the same assignments.

Figure 5a,b present a comprehensive examination of the location and strength of the peaks in the freshwater shell samples of *A. anatina* and *U. delicatus* within the intervals of 2500–3000 and 3000–4000 cm^–1^. Raman spectra shown in Figure 5a, the freshwater shell samples showed clear Raman peaks corresponding to C**–**H stretching vibrations, where the bands of variable intensity at 2835–2880 cm^–1^ (due to asymmetric stretching of ν­(CH_2_) in lipids and proteins) and 2909–2967 cm^–1^ (due to ν­(CH_3_) groups). The bands within the range of 2750–2780 cm^–1^ correspond to stretching vibrations of C–H, N–H, and −OH groups, while the amide I vibration band at 1620 cm^–1^, serves as an indication of protein molecules.^59–61^

The spectra presented in Figure 5a exhibit a notable resemblance in the bands of the two freshwater mussel shells. Additionally, Figure 5b illustrates the polarized Raman spectra within the 3000–4000 cm^–1^ range for both *A. anatina* and *U. delicatus* freshwater shell samples, together with the nonpolarized Raman spectrum of the corresponding shell sample. The Raman features observed in Figure 5b within the 3000–4000 cm^–1^ range are attributed to the stretching vibration modes of hydroxyl (−OH) groups and structural H_2_O molecules. The −OH vibration in the Raman spectra of *A. anatina* and *U. delicatus* freshwater shell samples manifests three prominent modes at approximately 3500/3483, 3573/3595, and 3645/3640 cm^–1^. The distinctive sharp asymmetric band for *U. delicatus* at around 3550 cm^–1^ and the weak band for *A. anatina* at around 3560 cm^–1^ are characteristic of the OH_3_ stretching vibration of in both samples.^62^ The minor shifts in the positions of all Raman spectra may be due to the effect of trace elements and natural impurities present in the samples.

The investigation of amyloid β (Aβ) peptides on ganglioside-GM1-containing lipid membranes by Hu et al.^59^ used fluorescence microscopy and Raman spectroscopy. The study found that in the Raman spectra, the ν­(C–H) region (2800–3000 cm^–1^), C–C vibration region (1000–1200 cm^–1^), and CH_2_ and CH_3_ stretching regions (2800–3000 cm^–1^) displayed peak frequencies and assignments consistent with those identified in this study for organic materials. Another detailed study on abdominal human skin and pig ear skin using confocal Raman micro spectroscopy at 660 nm, conducted by Tfaili et al.,^60^ identified significant differences in lipid content, hyaluronic acid, and carotenoid levels. Czamara et al.^61^ reported the Raman spectra of 35 lipids, including key lipids such as fatty acids, triacylglycerol’s, cholesterol, cholesteryl esters, and phospholipids, highlighting differences in Raman signatures within and between lipid groups. Both studies reported the same characteristic Raman band assignments between 2500 to 3000 and 3000 to 4000 cm^–1^, consistent with the findings of this study.

### Shell Microhardness

Microhardness examination of mussel shells was performed through microindentation on two individual shells, covering the horny layer, prismatic layer, and nacreous layer. The assessment encompassed two separate mussel shells, focusing on the three layers mentioned. Microhardness measurements were conducted in three categories, advancing from the inner layer to the outer layer along a cross-section roughly parallel to the mussel shells growth lines.

The microhardness values provided in Figure 6 represent the average of 12 measurements at each position along the shell’s thickness direction. The nacreous layer exhibits the highest microhardness value in both *A. anatina* and *U. delicatus* mussel shells, with respective values of 596 ± 21.74 Vickers hardness and 208.4 ± 5.23 Vickers hardness. In *A. anatina*, the horny layer demonstrates an average microhardness value of 575 ± 40.31 Vickers hardness, surpassing the prismatic layer, which records at 449.5 ± 32.18 Vickers hardness. Conversely, in *U. delicatus*, the mean microhardness value in the horny layer is 171.4 ± 15.9 Vickers hardness, which is less than the microhardness observed in the prismatic layer, measuring 196.5 ± 18.6 Vickers hardness. The differences in microhardness values among the three layers in mussel shells of *A. anatina* and *U. delicatus* are ascribed to their distinct microstructures, despite having similar phase components, as illustrated in Figure 6.

**Figure 6:**
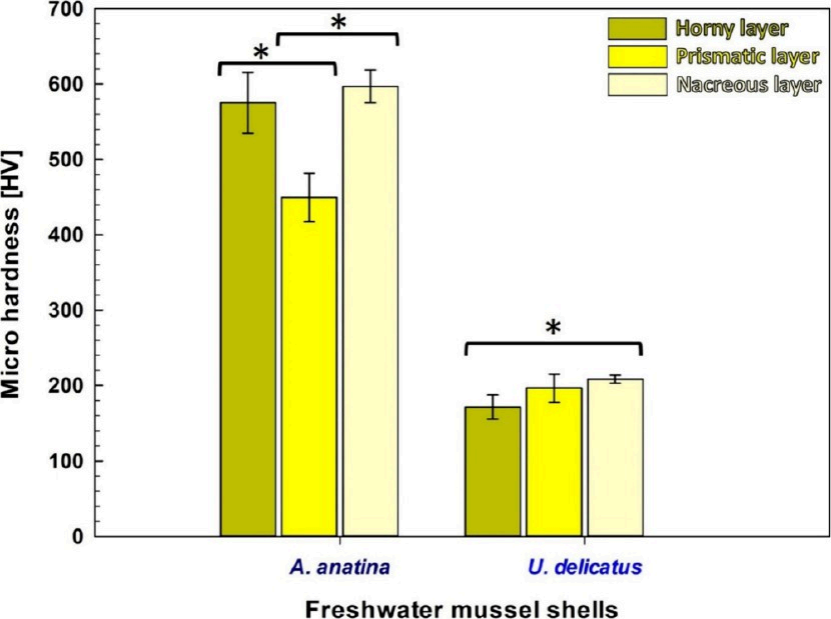
Microhardness properties of the shells, assessed through Vickers microhardness testing, were examined on cross-sectional surfaces of *A. anatina* and *U. delicatus*. The testing covered the horny layer, prismatic layer, and nacreous layer, and the mean values are depicted in the bar chart (mean ± SD, *n* = 12*,* *p ≤ 0.05).

### Morphology, Biostructure, and Materials Properties of Bivalves

The structural morphologies and surface topography of *A. anatina* and *U. delicatus* freshwater mussel shells were examined using a FE-SEM. This involves analyzing the surface texture, roughness, and any notable features such as ridges, pores, or patterns.

Both *A. anatina* and *U. delicatus* mussel samples exhibited oval-shaped shells with a white-creamy layer of yellow shell. However, *A. anatina* displayed a wide and shallow layer (Real photos in Figure 7), while *U. delicatus* had a thicker shell layer (Real photos in Figure 8). The outer shell (periostracum) of these species was coated with an irregular layer of chitin, which is a type of polysaccharide. On the inner surface, the shells appeared pure white and exhibited a smooth, glossy texture. The predominant segment of the internal surface of a bivalve is composed of the aragonitic nacreous layer, whereas the calcitic prismatic layer is observable on both surfaces of the shell. The dimensions of the clearly and unequivocally visible prismatic layers in the microstructure were measured as 26 ± 1.25 μm for *A. anatina* (Figure 7a) and approximately 30.75 ± 0.86 μm for *U. delicatus* (Figure 8a).

**Figure 7:**
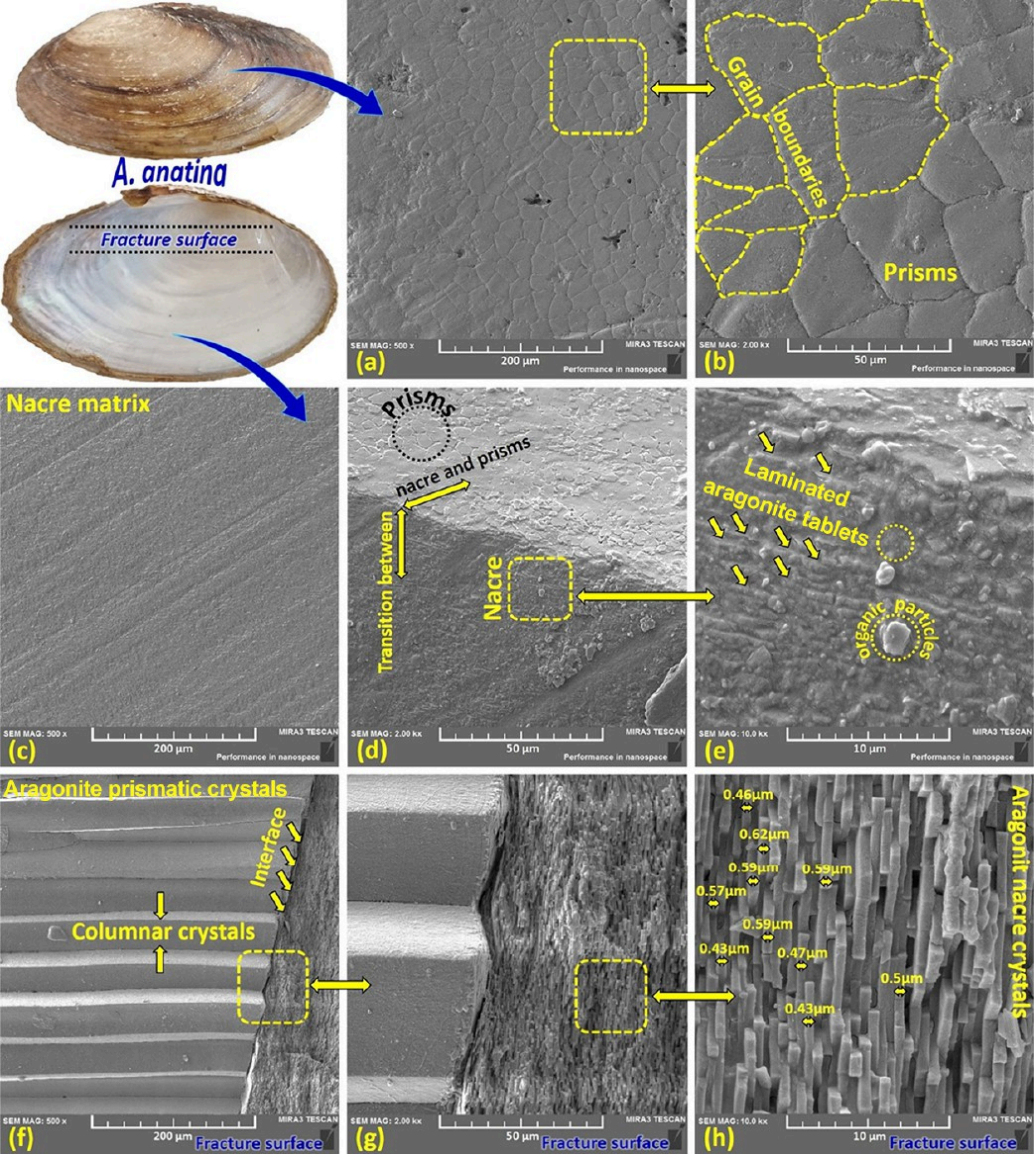
Real photos of *A. anatina* from the Gölbaşı Lake, Hatay, Türkiye. (Overall view of the shell showing its outer and inner surfaces). An examination of the *A. anatina* freshwater mussel shell provides insights into its microstructure. Microstructural view of *A. anatina* freshwater mussel shell showing the (a, b) outer prismatic layer (prisms). (c) Internal perspective of *A. anatina* revealing the inner nacreous layer (nacre). This layer exhibits distinct characteristics that contribute to the shell’s unique properties. (d) Transition zone: Significantly, there is a transition zone observed between the nacre and the outer prismatic layer (prisms). This region marks the interface between the two layers and presents its own distinctive features. (e) Magnified microstructure micrographs of the inner layer: By conducting a parallel section analysis, a magnified view of the outer-inner layer is obtained. This detailed examination reveals the presence of laminated aragonite tablets and organic particles within the nacreous layer. These components contribute to the intricate composition and functionality of the shell. (f) Cross-sectional view of the inner layer of *A. anatina* reveals its intricate composition, featuring an outer layer known as the periostracum, followed by a remarkable arrangement of aragonite tablets in a nacreous microstructure. Additionally, the transition zones between each of these layers exhibit unique properties that contribute to the shell’s overall strength and resilience. (g) Detailed microstructural orientation of the fracture surface reveals the presence of columnar crystals, specifically aragonite nacre crystals, along with distinct transition zones. (h) Overall views: Cross-sectional and in-plane FE-SEM micrographs depicting the microstructures of nacre in *A. anatina* shells.

**Figure 8:**
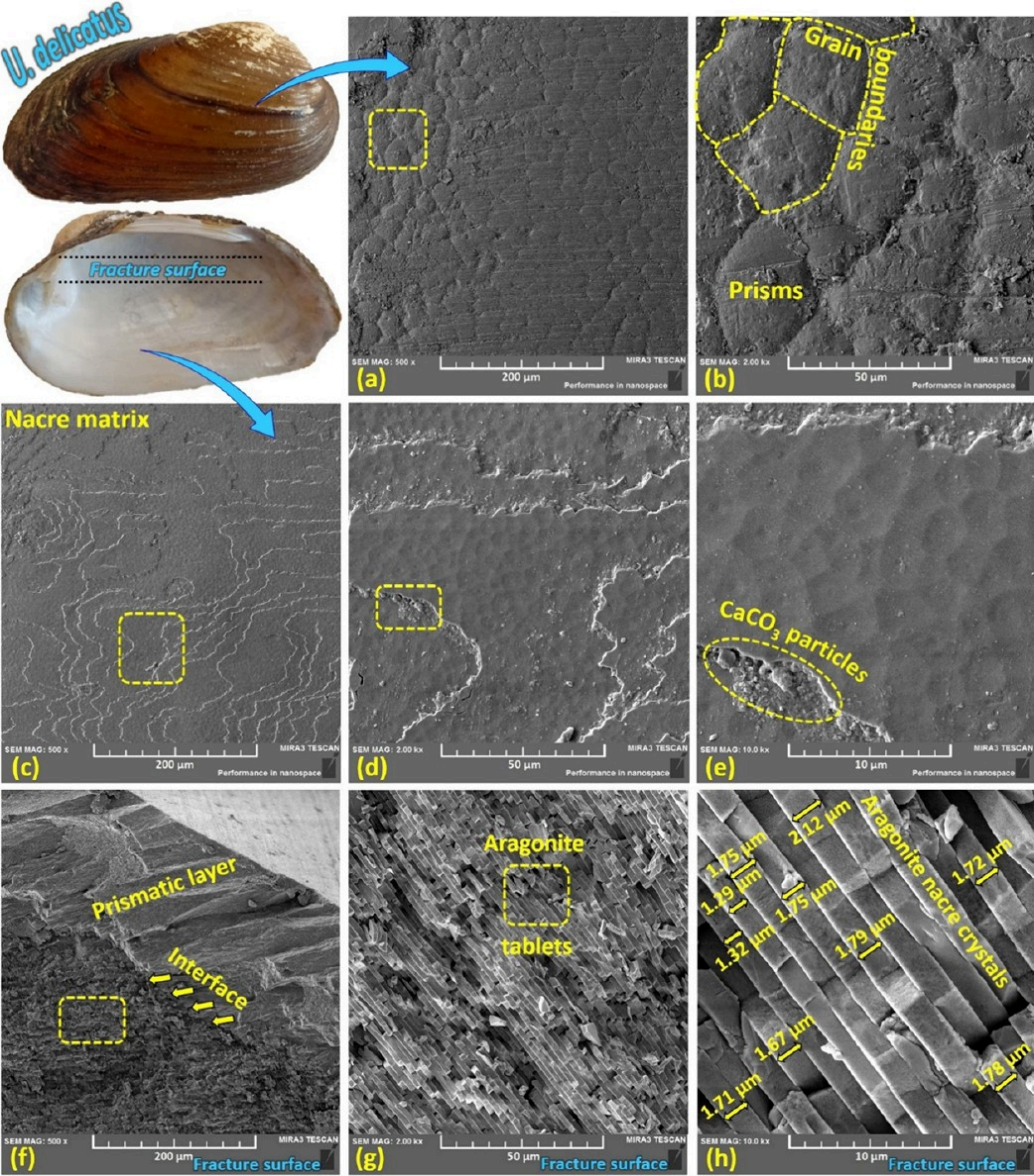
Real photos of *U. delicatus* from the Gölbaşı Lake, Hatay, Türkiye. (Overall view of the shell showing its outer and inner surfaces). Microstructural view of *U. delicatus* freshwater mussel shell showing the (a, b) outer prismatic layer (prisms). (c) Internal perspective of *A. anatina* revealing the inner nacreous layer (nacre). (d) Detailed microstructural orientation of the nacreous layer showing the shape of nacreous platelets as a staggered nacreous structure. The staggered nacreous structure, also known as a staggered brick-wall structure, represents a distinctive organization of platelets within the nacre (mother-of-pearl) layers of particular mollusk shells. In the staggered nacreous structure, the platelets are arranged in a brick-like pattern but with a staggered alignment. This means that each row of platelets is slightly offset from the row above and below it, resembling the arrangement of bricks in a brick wall. This staggered alignment is a key factor that contributes to the remarkable mechanical properties of nacre. (e) High-magnification view of the nacreous structure, showcasing the nacreous layers with (CaCO_3_)-based powders deposited between them. (f) Inner layer of *U. delicatus*, when examined in cross-section, unveils a complex composition, characterized by an outer layer identified as the periostracum, accompanied by a notable arrangement of aragonite tablets in a nacreous microstructure. (g) Examination of the fracture surface’s microstructural orientation provides clear evidence of the presence of aragonite nacre crystals. (h) Comprehensive perspectives, cross-sectional observations, and in-plane FE-SEM micrographs depict the high-magnification structures of nacre in *U. delicatus* shells.

The results obtained from the FE-SEM analysis showcased the excellent preservation of the nacreous layer in every bivalve species examined, as illustrated in Figures 7c–e and 8c–e. The nacreous layer, known as mother-of-pearl,^63^ is an internal lustrous layer found in various mollusks, including the mussel, pearl oyster, abalone, and nautilus. This layer, which is consistently composed of aragonite, possesses remarkable mechanical properties that make it an intriguing subject of study in mollusk shell microstructures.^64^ Its distinctive characteristics have attracted significant research attention due to its intrinsic strength and durability. Figures 7 f–h and 8f–h show the cross sections of the *A. anatina* and *U. delicatus* freshwater mussel samples, respectively. The prismatic structure of both freshwater mussels comprises columnar crystals that are interspersed with an organic framework, which acts as a binding material between the columnar crystals. Within the prismatic layer, there exist typically one or two layers of columnar crystals arranged perpendicularly to the shell surface. Conversely, the nacreous layer comprises thousands of layers of tablet-shaped crystals. It can be seen from the cross-section views in Figures 7f and 8f that the shells of the freshwater mussel contain different prismatic layers, each distinct from the other. In *U. delicatus*, the prismatic structure (columnar crystals) is formed by irregular aragonite fibers, creating a common structural module. In contrast, *A. anatina* exhibits distinct columnar prisms with simple aragonite prism fibers, arranged in a polygonal shape. Below, we present a schematic illustration of the prismatic structure at the mesoscale, showcasing two different vertical/distinct and complex/nondistinctive columnar crystal structures in *A. anatina* and *U. delicatus* freshwater mussel species respectively. Significant differences are observed in terms of fiber length and orientation, which play a vital role in shell growth and remodeling.^65,66^ The fiber lengths measured were 198.3 ± 9.960 μm for *U. delicatus* and 494.3 ± 7.665 μm for *A. anatina*, highlighting the substantial disparity between the two species.

The transition between the prismatic and the nacreous layer in *A. anatina* is clearly evident, displaying a sharp and distinct separation (Figure 7f). The demarcation line between the prismatic layer and the nacreous layer in *A. anatina* is notably well-defined, with a clear contrast in the arrangement and composition of the respective layers. This distinct separation indicates a marked difference in the crystal orientation and organization between the prismatic and nacreous regions. Upon examining the microstructure of *U. delicatus*, it was observed that the transition between these layers was distinguishable, although not as sharply distinct as in *A. anatina* (Figure 8f). In the case of *U. delicatus*, while there is a discernible transition between the prismatic and nacreous layers, the distinction is not as sharp as observed in *A. anatina*. The boundary between these layers shows some overlapping characteristics, suggesting a more gradual shift in crystal structure and composition.

### Hierarchical Structure of Nacre Layers

Nacre exhibits a highly intricate hierarchical structure that extends across a range of length scales, extending from the nanoscale to the macroscale,^67^ as schematically represented in Figure 9a,b. This remarkable architecture is not only limited to nacre but is also a significant feature observed in structural materials and soft tissues present in mammals, including bones, teeth, and other bodily tissues.^68^ When comparing the nacre of different freshwater mussel species, namely *A. anatina* and *U. delicatus*, several notable distinctions were observed. The thickness of the nacreous platelets exhibited slight variations across the species under investigation. In most cases, the thickness was approximately 0.52 ± 0.07 μm or less for *A. anatina*. However, when comparing it to *U. delicatus* (also as shown in Figures 7 and 8), a significant difference in the thickness of individual nacreous platelets was detected. Specifically, *U. delicatus* exhibited a noticeably greater thickness of 1.707 ± 0.14 μm, surpassing the measurements recorded for *A. anatina*.

**Figure 9:**
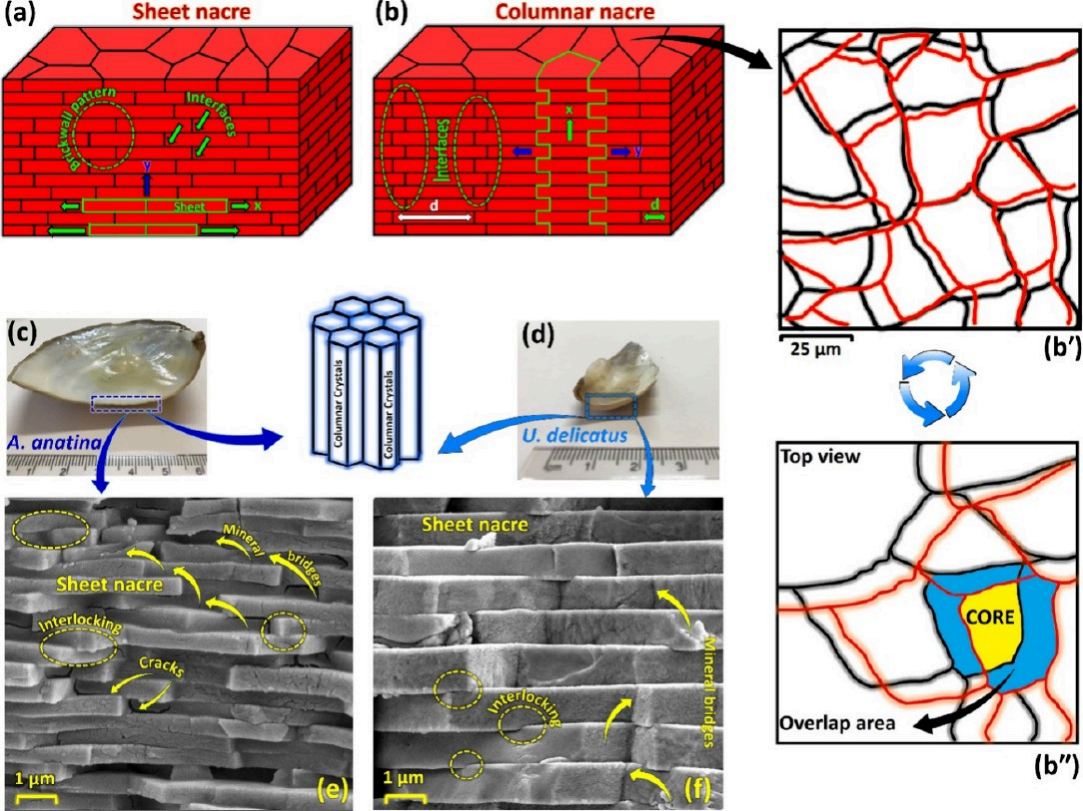
Schematic illustrations of the microstructures in (a) sheet and (b) columnar nacres. Cross-sectional FE-SEM micrographs of (c) *A. anatina* and (d) *U. delicatus* freshwater mussel shells. The FE-SEM micrographs indicate that the prismatic layer of both bivalves primarily consists of columnar crystals incorporated in an organic matrix. High-resolution FE-SEM micrographs provide a detailed view of the nacreous platelets in both (e) *A. anatina* (sheet nacre) and (f) *U. delicatus* bivalves (sheet nacre). Additionally, the FE-SEM micrographs capture perpendicular nacre platelets, revealing their interlocking structures, mineral bridges, and cracks within each individual shell. The figure presents schematic illustrations depicting the distinct microstructures found in (e, f) sheet nacres. (b́) Top view of the columnar nacre microstructure, illustrating the reconstruction of tablet arrangements from one layer to the next. This reconstruction helps to understand how the organization and alignment of the tablets evolve throughout the nacreous layers, elucidating the nucleation and growth processes involved in creating these remarkable structures. (b́́) Detailed representation of nucleation cores and overlap regions within the tablet configurations. The analysis of these overlap areas demonstrates how neighboring tablets interact to enhance the overall strength and resilience of the nacreous structure in the biomineralized structure.

In order to establish the essential microstructural traits of nacre in both *A. anatina* and *U. delicatus* freshwater mussel species, it is imperative to initially elucidate the specific mechanisms underlying their growth. The impressive mechanical attributes of nacre can be ascribed, at least in part, to the meticulous geometric organization of its individual tiles.^69^ This is because the structural variances observed between sheet and columnar nacres offer significant clues into their unique modes of growth. To elucidate the growth mechanisms inherent to gastropods and bivalves, it is essential to characterize their microstructural features at the microscale. The structural variances observed between sheet nacre and columnar nacre explain into their distinct modes of growth. It is generally established that columnar nacre typically forms within a narrow zone at the margins of certain gastropods, whereas sheet nacre is deposited across the majority of the inner shell surface, a pattern predominantly observed in bivalves.^64^ Initially, crystals in gastropods are vertically oriented (indicated by the *x*-direction in Figure 9b), arranged in a stacked manner resembling a “*stack of coins*” structure.^70^ The crystals then undergo lateral growth until a continuous crystal layer is formed (represented by the *y*-direction in Figure 9b), resulting in the final nacreous layer.^71^ The deposition process in question varies from that observed in bivalves, where aragonite crystals are deposited layer by layer. In bivalves, nacre grows in a terraced fashion, with each successive layer expanding horizontally on top of the preceding one (initially along the *x*-direction and subsequently the *y*-direction, as illustrated in Figure 9a).^71,72^ As a result of these unique characteristics, nacre showcases an intriguing aragonite laminar structure. It is composed of individual polygonal or rounded platelets that intricately interlock with each other, giving rise to two distinctive forms: columnar nacre and sheet nacre. The specific arrangement in which the platelets stack determines whether the resulting nacre structure assumes a columnar or sheet-like configuration. This stacking mode is visually represented in Figure 9a,b, illustrating the intricate interplay and alignment of the platelets. This intricate interlocking arrangement contributes to the remarkable strength and resilience exhibited by nacre, making it a highly sought-after biomaterial in various fields of study and biomedical applications. Accordingly, when examining the cross-section of nacre, notable distinctions can be observed. In sheet nacre, the platelets exhibit a staggered arrangement resembling a *brick wall* structure. On the other hand, columnar nacre can consist of stacks of aligned platelets, with some columns containing hundreds of platelets aligned together (as shown in Figure 9a,b). When observing mature columnar nacre from the top, the platelets appear uniform in size, displaying clearly defined nucleation cores and overlap regions (as depicted in Figure 9b′,b″). However, in sheet nacre, there is no apparent differentiation between the nucleation cores and overlap areas.^64^ The differentiation between the core and overlap region holds significance in columnar nacre, as these two areas undergo varying levels of stress.^73^ These variations in growth and nucleation mechanisms result in distinct stacking patterns. Within columnar nacre, platelets are arranged in stacks along the columns, featuring specific overlap regions between adjacent layers. Conversely, sheet nacre presents a *brick wall* pattern where platelets extend across the interface between underlying layers.^74,75^

Furthermore, FE-SEM analysis of the fractured nacreous surfaces of both species confirmed the sheet nacre configuration, characterized by intricate interlocking as shown in Figure 9e,f. These observations indicate that the brick-like platelets in nacre not only arrange themselves in a brick wall structure but also penetrate and interlock with one another. The presence of such interlocking mechanisms within the nacre structure serves as a crucial factor in imparting exceptional toughness and strength to the material. These interlocks act as physical barriers, preventing the mineral platelets from moving relative to one another. Consequently, these interlocks must fracture before fully transferring the load to the organic matrix.^76^ In addition, these interlocking properties are likely to play an important role in inhibiting the propagation of cracks through mechanisms such as crack branching and blunting, thereby increasing the overall fracture resistance of nacre.^77^ The interlocking nature of the platelets in nacre highlights their crucial contribution to the remarkable mechanical properties exhibited by this biomaterial.

### EDXS Analyses

The elemental composition on the surface of the *A. anatina* and *U. delicatus* freshwater mussel samples was examined through FE-SEM-EDXS spectra. The EDXS spectra, along with their corresponding calculated values, are presented in Figure 10. As depicted in Figure 10, the EDXS spectra revealed that both samples were predominantly composed of calcium, carbon, and oxygen, affirming the presence of these components in the freshwater mussel shell samples. Additionally, while sodium was detected as a secondary constituent in the *A. anatina* samples, its presence in *U. delicatus* was negligible or below the detection threshold. The EDXS results for *A. anatina* revealed inclusion peaks of carbon and sodium within the aragonite (CaCO_3_) phase, supporting the observed differences in morphology in comparison to *U. delicatus* freshwater mussel samples as seen in the FE-SEM and XRD results.

**Figure 10:**
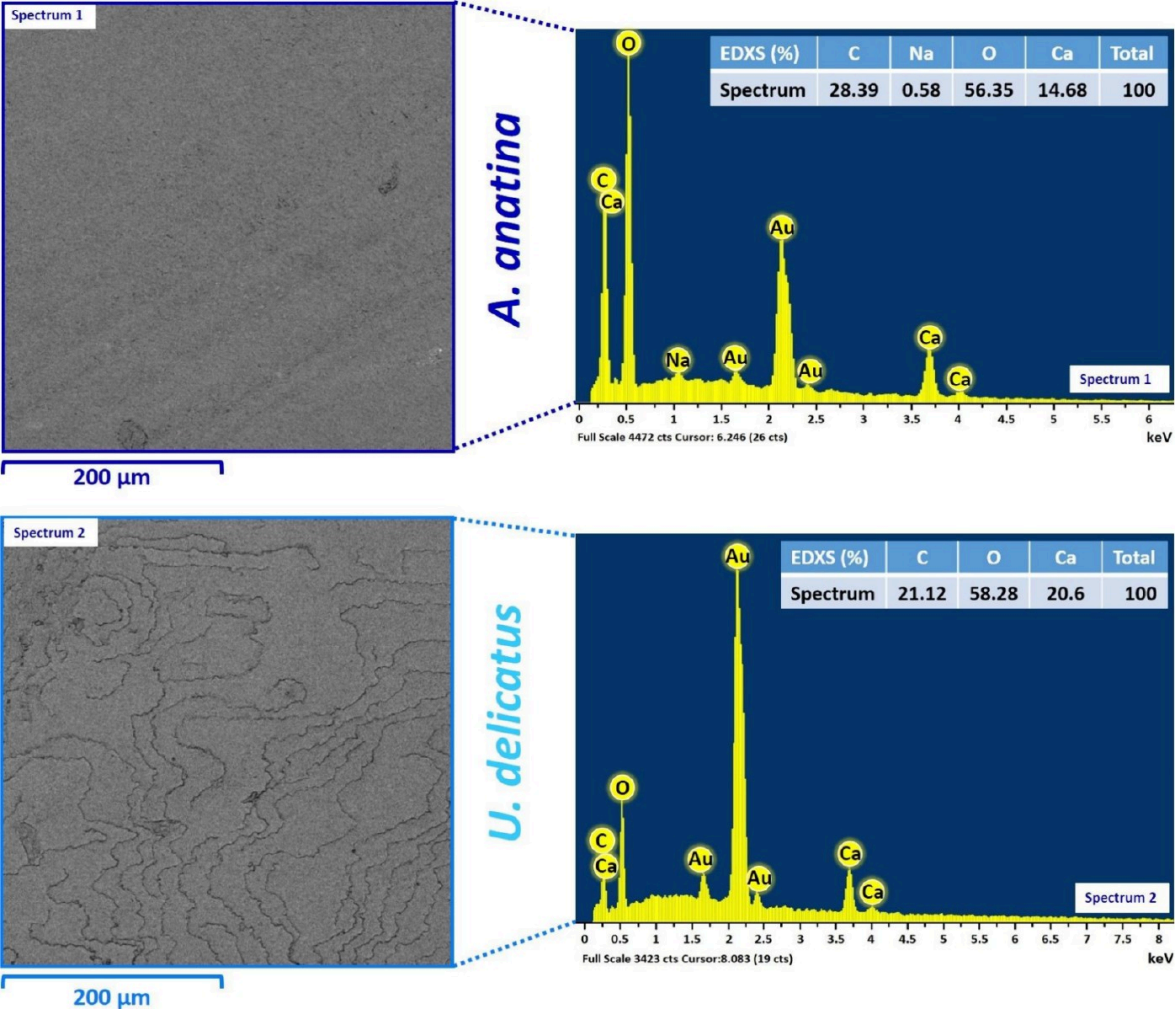
EDXS spectra derived from FE-SEM micrographs of the surfaces of both freshwater mussel shell samples offer details about the elements present on the surface and their distribution. A comparison of these spectra aids in evaluating variations in the elemental composition of the shell surface among different species.

### In Vitro Cytotoxicity Assessment

Assessing biocompatibility is a vital aspect of biomaterial development, which pertains to the biomaterial’s capacity to coexist with living tissue without inducing any detrimental effects. The biocompatibility of biomaterials is typically assessed through in vitro examinations. Among the most commonly employed in vitro assessments for biocompatibility is the cytotoxicity assay. This assessment involves exposing the material to living cells, often in a cultured environment, to ascertain if it elicits any adverse responses in the cells. Cytotoxicity tests play a pivotal role in biomaterial evaluation, as they offer insights into the material’s potential toxicity, contributing to the assurance of patient safety.

To investigate the cell proliferation of mouse fibroblast L929 cells on *A. anatina* and *U. delicatus* shell samples, cell viability. was assessed using the XTT assay with dosages of 2, 4, 6, and 10 μL. Figure 11a shows the schematic representation of in vitro XTT assay test steps, and Figure 11b presents the cell viability plot obtained from the XTT assay, indicating that each group facilitated cell proliferation, maintaining cell viability values consistently exceeding around 76.65%. The XTT cytotoxicity study indicated that both the *A. anatina* and *U. delicatus* shell samples were nontoxic. As shown in Figure 11b, L929 cells cultured on *U. delicatus* shell samples exhibited higher viability compared to those on *A. anatina* shell samples, particularly in the case of high dosage applications (6 and 10 μL), implying excellent biocompatibility. The XTT assays show a significant difference in cell activity between the *U. delicatus* group (94.10% ± 0.91, 2 μL; 77.01% ± 0.74, 4 μL; 93.88% ± 0.98, 6 μL) and the *A. anatina* group (85.4% ± 0.83, 2 μL; 76.65% ± 0.68, 4 μL; 97.69% ± 0.92, 6 μL) compared to the control group, indicating their potential to support cell growth. At the highest dosage application (10 μL), the cell viability was found to be 90% ± 0.8 for the *A. anatina* group and 93.47% ± 0.83 for the *U. delicatus* group. Furthermore, cell viability changes with the increase in applied dosage, demonstrating that the incorporation of trace elements through dissolution has a positive effect on the excellent biocompatibility of shell samples.

**Figure 11:**
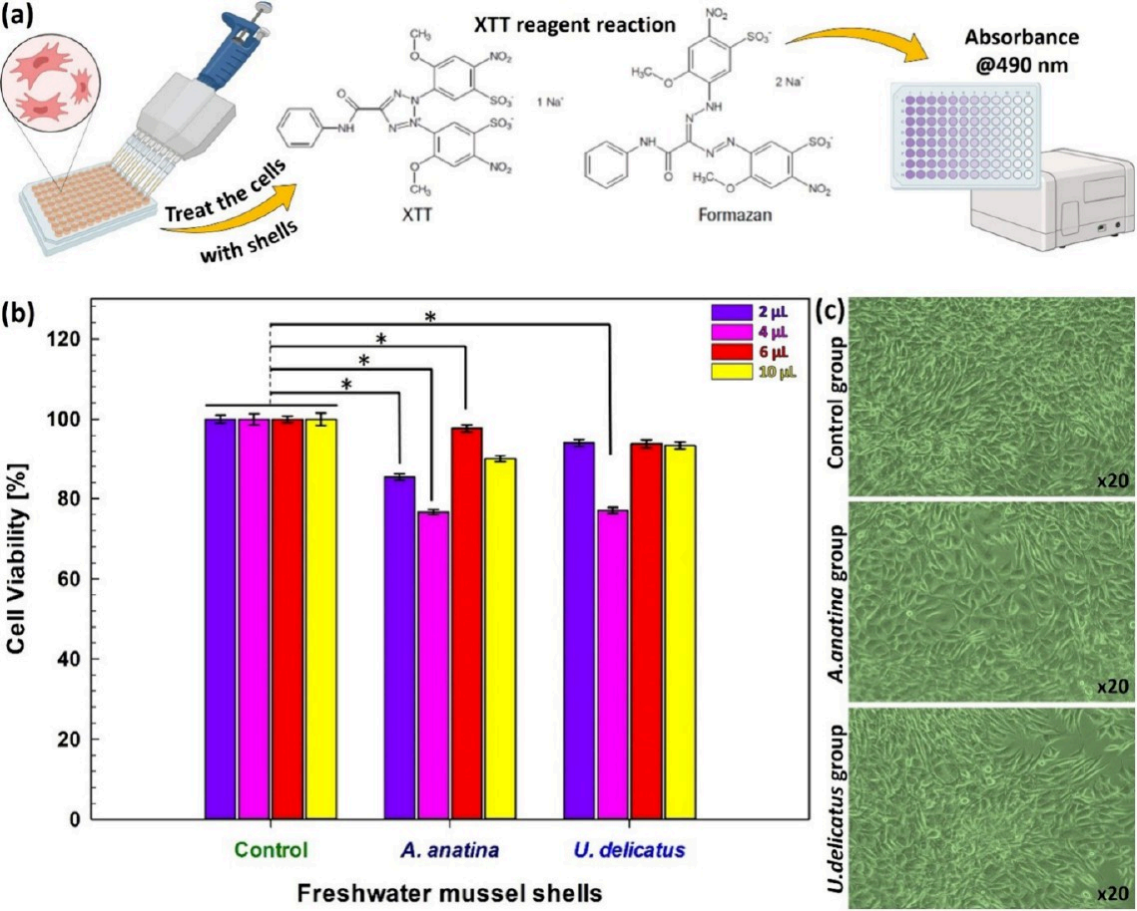
(a) Visual representation of an in vitro assay conducted using mouse fibroblast L929 cells, specifically designed to assess the cell viability of both the *A. anatina* and *U. delicatus* groups. (b) This diagram illustrates cell viability percentages determined through the XTT assay for each group. The values presented here represent the mean cell viability, accompanied by the standard deviation (±SD), based on the results of three distinct and independent experiments. Significance levels were assessed, with statistical analysis revealing significant differences between groups (*p < 0.05). (c) Figure vividly illustrates the notable morphological changes in L929 cells following a 24-h treatment at 37 °C. These transformations were meticulously observed under a high-quality inverted microscope, with a powerful 20× magnification. Notably, the L929 cells displayed a striking fusiform morphology, characterized by their elongated, spindle-shaped appearance. This fusiform morphology holds distinct advantages, as it is well-suited for studies involving cell motility and migration, making it an ideal model for investigating cellular responses to various treatments and environmental conditions.

Mollusk shells are known to comprise various macromolecules, such as soluble proteins, glycoproteins, chitin, lipids. and soluble polysaccharides.^63,78^ Extracts from *Potomida semirugata* and *Leguminaia wheatleyi* shells have been reported in the literature^79^ to promote cell proliferation, and there is a suggestion that shell proteins might contribute to this observed effect. To further confirm the biocompatibility of both shell samples, the morphology of L929 cells grown on the control group, *A. anatina* group, and *U. delicatus* group was observed using an inverted microscope (Olympus BX51, Japan) after 24 h of culture. The cell profiles (for 10 μL) in both the control group and the *A. anatina* and *U. delicatus* group samples are clearly visible (Figure 11c). Notably, the cells in both groups exhibit a typical fusiform shape, and the strong interaction between the cells and shell samples is evident.

### Hemolysis Assay and Hemostasis Studies

The hemocompatibility of the *A. anatina* and *U. delicatus* freshwater mussel shell samples was evaluated by employing an in vitro hemolysis and blood clotting tests. Figure 12a,b depicts the schematic illustration of in vitro hemolysis/blood clotting assay steps, while Figure 12c,d displays the results of hemolytic activity (HA) and blood clotting index (BCI) for *A. anatina* and *U. delicatus* freshwater mussel shell samples using fresh blood. Following a 2 h incubation period at 37 °C, the visible color of the mussel shell samples, along with the positive control group (comprising RBCs in dH_2_O at a 1:4 mL ratio), was examined. It was observed that all the shell sample groups exhibited a pale yellow coloration, in contrast to the vivid bright red coloration of the positive control group. This divergence in coloration suggested that the extent of hemolysis might vary between the different groups. The hemolytic activity was 1.49% ± 0.008 and 2.29% ± 0.183 for the *A. anatina* and *U. delicatus* groups, respectively (as depicted in Figure 12c), indicating that the hemolytic activities slightly changed with the different species. The differences in HA values can be attributed to the varying compositions of the mussel shells of the two species. The shells may have different physical properties, such as particle size, structure, or chemical composition, which can affect their interactions with RBCs and the degree of hemolysis.^80^ When assessing and categorizing the hemolytic properties of different biomaterials intended for healthcare applications, the prevailing standards commonly acknowledged are those established by the American Society for Testing and Materials (ASTM). According to the ASTM guidelines, particularly ASTM F756-00, as well as ISO standards 10993–5:1992, a sample is deemed nonhemolytic if its HI falls below 2%. If the HI ranges between 2 and 5%, the biomaterial is categorized as mildly hemolytic, whereas any percentage exceeding 5% designates the biomaterial as hemolytic.^81^

**Figure 12:**
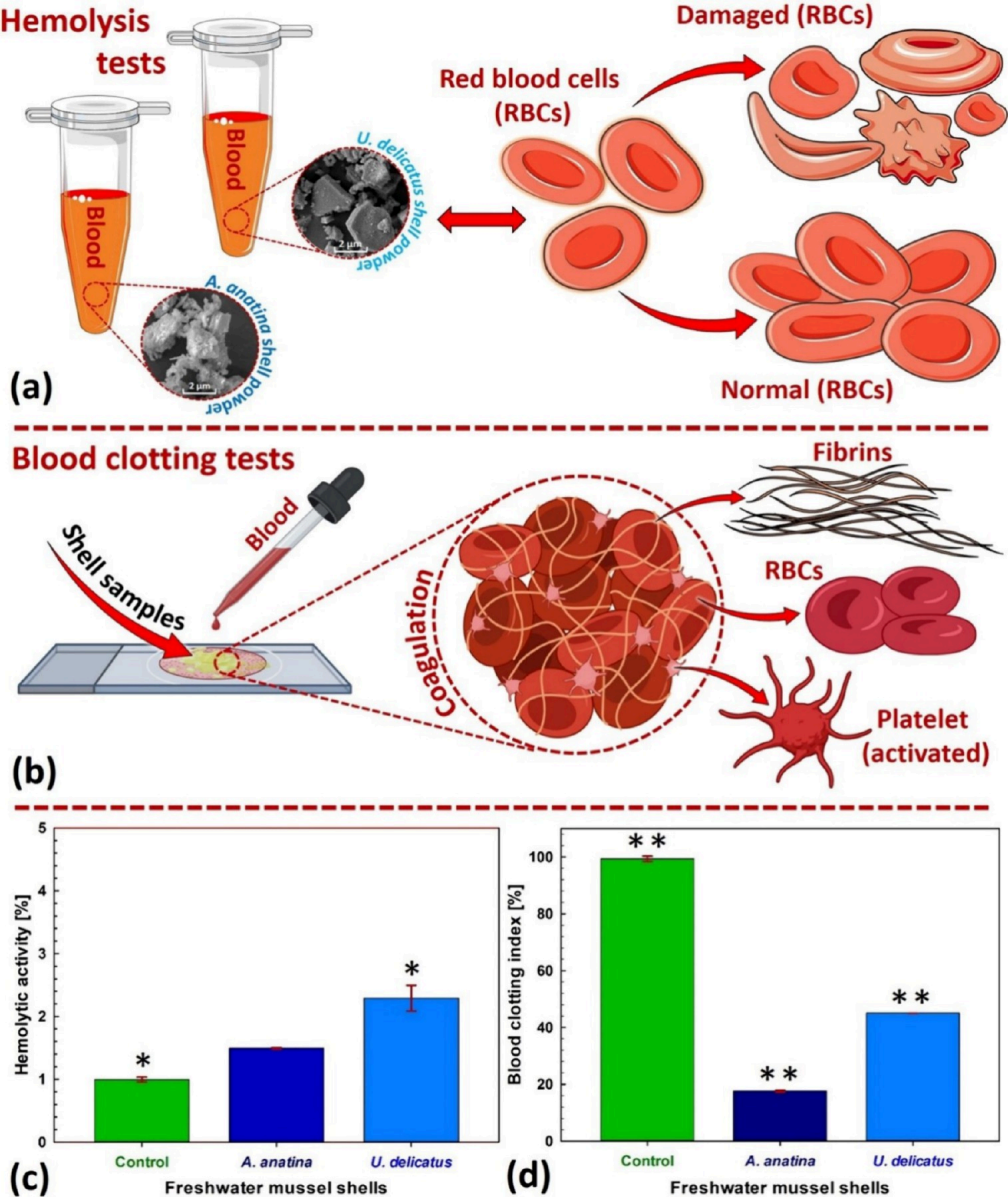
(a) Schematic illustration of in vitro hemolysis assay steps and (b) hemostasis (blood clotting) process of *A. anatina* and *U. delicatus* shell samples with fresh blood. This detailed schematic presentation provides an in-depth visualization of the procedural steps involved in the in vitro hemolysis test. Furthermore, it elucidates the complex and fascinating process of hemostasis, which encompasses the intricate mechanisms of blood clotting. (c) In vitro hemolytic activity of *A. anatina*, *U. delicatus*, and the control group, as well as (d) in vitro blood clotting index of *A. anatina*, *U. delicatus*, and the control group. The values presented indicate the mean and ±SD derived from three independent experiments (*n* = 3). (*p < 0.05; **p < 0.001 *Statistically significant differences between groups).

The coagulation potential of freshwater mussel shells was examined by assessing their ability to induce blood clotting in an in vitro whole blood clotting assay. The level of blood-hemostat clot formation is typically quantified using the BCI. A lower BCI value signifies a greater blood clotting activity. As depicted in Figure 12d, the BCI for the control group was 98.75% ± 0.183. Notably, the BCI values for the *A. anatina* and *U. delicatus* groups sharply decreased to 17.614 ± 0.28 and 45.03% ± 0.02, respectively. These findings illustrate that *A. anatina* freshwater mussel shell samples accelerate the clotting process, with *A. anatina* shells exhibiting the most effective promotion of coagulation. Coagulation activation is closely linked to the role of cationic elements in the process, as it aids in complex binding and the activation of specific factors. Cationic materials can attract RBCs and platelets, leading to the acceleration of the coagulation process. This dynamic interplay between cationic elements and blood components not only enhances the clotting mechanism but also ensures a swift and efficient response when needed, contributing to the overall effectiveness of coagulation in maintaining hemostasis and wound healing.^82,83^

## Conclusions

The viability and longevity of freshwater mussels hinge extensively upon the intricate microstructures, robust resistance properties, and the diverse material components embedded within the composition of their shells. These elements collectively play a pivotal role in ensuring the resilience and adaptability of freshwater mussels to their dynamic aquatic environments, contributing substantially to their overall survival in various ecological settings. In this paper, the crystalline organization, hierarchical structure, mechanical resistance, shell components and biological responses of the freshwater mussel shells of *A. anatina* and *U. delicatus* were investigated. Through the application of FT-IR analysis, the investigation into shell samples revealed the presence of B-type CO_3_^2–^ and aragonite. The XRF data further elucidated that the predominant constituent of the mussel shell biofiller is CaCO_3_. Upon comparing the XRD patterns of shells originating from two distinct mussel specimens, notable resemblances in crystalline peaks were observed. This observation revealed that the coexistence of both aragonite and calcite forms of CaCO_3_ in the mussel shells. To further explore the molecular composition, Raman spectroscopy was employed as a method to pinpoint specific molecules or functional groups present in the shells, providing insights into their characteristic vibrational frequencies. The microstructure of the shells displayed a uniform surface design, interface architecture and a systematic organization of crystal fibers across the entire framework. Shells with elevated mineral content and a nacreous layer exhibited impressive mechanical strength as a biological material. The biomineralization process, involving growth and remodeling, leads to the development of a layered pattern of crystalline aggregates in mussel shells. This pattern ensures both structural and functional adaptability, as well as stability, to uphold the structural hierarchy.

XTT cytotoxicity analysis indicated nontoxic characteristics for both shell samples. The hemocompatibility properties of both mussel shells were confirmed to be within acceptable limits, and *A. anatina* shell samples were found to accelerate the coagulation process and support coagulation in the most effective way. Hence, the outcomes derived from experimental results conducted on the shells of unionid mussel species such as *A. anatina* and *U. delicatus* freshwater mussels hold the potential to ensure valuable insights and guidance for the development of biomimetic materials. These findings may serve as a foundation for novel approaches in the design and application of biomaterials inspired by the unique characteristics and properties observed in these particular mussel species.

## Supplementary Material



## Data Availability

The datasets produced in this study can be obtained from the corresponding author upon reasonable request.

## Notes

Highlights graphical abstract (PDF)

## References

[1] Vaughn, C. The functional ecology of freshwater mussels; Johns Hopkins University Press: Baltimore, 2025. 10.56021/9781421453521.

[2] Zieritz A., Froufe E., Bolotov I., Gonçalves D. V., Aldridge D. C., Bogan A. E., Gan H. M., Gomes-Dos-Santos A., Sousa R., Teixeira A., Varandas S., Zanatta D., Lopes-Lima M. (2021). Mitogenomic phylogeny and fossil-calibrated
mutation
rates for all F- and M-type mtDNA genes of the largest freshwater
mussel family, the Unionidae (Bivalvia). Zool.
J. Linn. Soc..

[3] Şereflişan H., Şereflişan M., Soylu S. (2009). Description of glochidia
of three species of freshwater mussels (Unionidae) from Southeastern
Turkey. Malacologia.

[4] Modesto V., Ilarri M., Souza A. T., Lopes-Lima M., Douda K., Clavero M., Sousa R. (2018). Fish and mussels: Importance
of fish for freshwater mussel conservation. Fish Fish..

[5] Şereflişan H. (2021). Host selection
of *Potomida semirugata* (Unionidae: Bivalvia) in reproduction
strategy. Aquat. Sci. Eng..

[6] Şereflişan, M. Threat factors of freshwater mussels. In Molluscs of Türkiye: Freshwater Bivalvia; Yildirim, M. Z. , Ed.; İksad Publishing House: Türkiye; Vol. 1, pp 139–156.

[7] Tomilova A. A., Zubrii N. A., Kondakov A. V., Vikhrev I. V., Gofarov M. Y., Lyubas A. A., Konopleva E. S., Chelpanovskaya O. A., Kruk D. V., Kebapçı Ü., Pokrovsky O. S., Bolotov I. N. (2025). Environmental, genetic, and age-induced
control explains
the continent-scale shell variability in a short-lived freshwater
mussel. Hydrobiologia.

[8] Liu C., Zhang W., Dong Q., Liu H. (2024). Exoskeleton protein
repertoires in decapod crustaceans revealed distinct biomineralization
evolution with molluscs. J. Proteomics.

[9] Perfetto R., Del Prete S., Vullo D., Sansone G., Barone C., Rossi M., Supuran C. T., Capasso C. (2017). Biochemical characterization
of the native α-carbonic anhydrase purified from the mantle
of the Mediterranean mussel, Mytilus galloprovincialis. J. Enzyme. Inhib. Med. Chem..

[10] Lopes-Lima M., Bolotov I. N., Do V. T., Aldridge D. C., Fonseca M. M., Gan H. M., Gofarov M. Y., Kondakov A. V., Prié V., Sousa R., Varandas S., Vikhrev I. V., Teixeira A., Wu R. W., Wu X., Zieritz A., Froufe E., Bogan A. E. (2018). Expansion and systematics
redefinition of the most
threatened freshwater mussel family, the Margaritiferidae. Mol. Phylogenet. Evol..

[11] Lopes-Lima M., Sousa R., Geist J., Aldridge D. C., Araujo R., Bergengren J., Bespalaya Y., Bódis E., Burlakova L., Van Damme D., Douda K., Froufe E., Georgiev D., Gumpinger C., Karatayev A., Kebapçi M., Killeen I., Lajtner J., Larsen B. M., Zogaris S. (2016). Conservation status of freshwater
mussels in Europe:
state of the art and future challenges. Biol.
Rev..

[12] Şereflişan H., Kebapçı Ü. (2025). Mineral
content and seasonal variation
of proximate composition in four sympatric freshwater mussels. Molluscan Res..

[13] Öksüz K.
E., Şereflişan H. (2022). Microstructure
of Eobania vermiculata
(Müller, 1774): SEM, F-TIR and XRD Methods. JAPRO.

[14] Öksüz K. E., Şereflişan H. (2023). Investigation of the structure and
hardness properties of Anodonta anatina mussel shells. EgeJFAS.

[15] Gao Z., Yang H., Liu Q. (2019). Natural seashell waste as an efficient
and low-cost catalyst for the synthesis of arylmethylenemalonitriles. Clean Soil Air Water.

[16] Öksüz K. E., Kilinç S., Özer A. (2019). Effect of calcination on microstructure
development and properties of hydroxyapatite powders extracted from
human and bovine bones. T. Indian Ceram Soc..

[17] Öksüz K. E. (2023). al_2_o_3_ particle size effect on reinforced Cu composites
produced by high energy milling. ADYUMBD.

[18] Öksüz K. E., Şen Ş., Şen U. (2018). Investigations on microstructural
and raman scattering properties of B2O3 doped Ba­(Ti1-xZrx)­O3 ceramics. J. Chem. Res..

[19] Dickinson G. H., Ivanina A. V., Matoo O. B., Pörtner H. O., Lannig G., Bock C., Beniash E., Sokolova I. M. (2012). Interactive
effects of salinity and elevated CO2 levels on juvenile eastern oysters,
Crassostrea virginica. J. Exp. Biol..

[20] Özer A., Öksüz K. (2019). The effect
of yttrium oxide in hydroxyapatite/aluminum
oxide hybrid biocomposite materials: Phase, mechanical and morphological
evaluation. Materialwiss. Werkstofftech..

[21] Prezant R. S., Dickinson G. H., Chapman E. J., Mugno R., Rosen M. N., Cadmus M. B. (2022). Comparative assessment of shell properties in eight
species of cohabiting unionid bivalves. FMBC.

[22] Meng Y., Guo Z., Fitzer S. C., Upadhyay A., Chan V. B. S., Li C., Cusack M., Yao H., Yeung K. W. K., Thiyagarajan V. (2018). Ocean acidification
reduces hardness and stiffness of the Portuguese oyster shell with
impaired microstructure: a hierarchical analysis. Biogeosciences.

[23] El-Bassyouni G. T., Eldera S. S., Kenawy S. H., Hamzawy E. M. (2020). Hydroxyapatite nanoparticles
derived from mussel shells for in vitro cytotoxicity test and cell
viability. Heliyon.

[24] Öksüz K.
E., Özkaya N. K., İnan Z. D. A., Özer A. (2021). Novel natural
spider silk embedded electrospun nanofiber mats for wound healing. Mater. Today Commun..

[25] Kurt B., Oksuz K. E., Sahin-Inan Z. D., Hepokur C. (2023). Efecto antiadhesivo
de nanopartículas de dihidrato de fosfato dicálcico
obtenidas naturalmente en el modelo de herida uterina de rata. Cir. Cir..

[26] Öksüz K. E., Kurt B., Şahin İnan Z. D., Hepokur C. (2023). Novel bioactive
glass/graphene oxide-coated surgical sutures for soft tissue regeneration. ACS Omega.

[27] Bohorquez-Moreno C. D., Öksüz K. E., Dinçer E. (2023). Porous polymer
scaffolds derived from bioresources for biomedical applications. Cellul. Chem. Techno..

[28] Wang S., Castro R., An X., Song C., Luo Y., Shen M., Tomás H., Zhu M., Shi X. (2012). Electrospun
laponite-doped poly­(lactic-co-glycolic acid) nanofibers for osteogenic
differentiation of human mesenchymal stem cells. J. Mater. Chem..

[29] Patil P. P., Meshram J. V., Bohara R. A., Nanaware S. G., Pawar S. H. (2018). ZnO nanoparticle-embedded
silk fibroin–polyvinyl alcohol composite film: a potential
dressing material for infected wounds. New J.
Chem..

[30] Dhanaraj K., Suresh G. (2018). Conversion of waste sea shell (Anadara granosa) into
valuable nanohydroxyapatite (nHAp) for biomedical applications. Vacuum..

[31] Seesanong S., Boonchom B., Chaiseeda K., Boonmee W., Laohavisuti N. (2021). Conversion
of bivalve shells to monocalcium and tricalcium phosphates: An approach
to recycle seafood wastes. Materials.

[32] Wong S., Eaton A., Krywka C., Nair A., Drouet C., Deymier A. (2025). Increasing A-type CO32-
substitution decreases the
modulus of apatite nanocrystals. J. Mech. Behav.
Biomed. Mater..

[33] Ren F., Ding Y., Leng Y. (2014). Infrared spectroscopic characterization
of carbonated apatite: A combined experimental and computational study. J. Biomed. Mater. Res., Part A.

[34] Tkalčec E., Popović J., Orlić S., Milardović S., Ivanković H. (2014). Hydrothermal
synthesis and thermal evolution of carbonate-fluorhydroxyapatite
scaffold from cuttlefish bones. Mater. Sci.
Eng., C.

[35] Giordano L., Ferraro L., Caroppo C., Rubino F., Buonocunto F., Maddalena P. (2022). A method for bivalve shells characterization by FT-IR
photoacoustic spectroscopy as a tool for environmental studies. Methods X.

[36] Pokrovsky O. S., Mielczarski J. A., Barres O., Schott J. (2000). Surface speciation
models of calcite and Dolomite/Aqueous solution interfaces and their
spectroscopic evaluation. Langmuir.

[37] Ramseyer K., Miano T. M., D’orazio V., Wildberger A., Wagner T., Geister J. (1997). Nature and origin of
organic matter
in carbonates from speleothems, marine cements and coral skeletons. Org. Geochem..

[38] Stanienda-Pilecki K. (2019). The importance
of Fourier transform infrared spectroscopy in the identification of
carbonate phases differentiated in magnesium content. Spectroscopy.

[39] Li S., Wang Z. J., Chang T. T. (2014). Temperature oscillation modulated
self-assembly of periodic concentric layered magnesium carbonate microparticles. PLoS One.

[40] Mei Y., Wang Z., Fang H., Wang Y., Huang J., Fang Y. (2017). Na-containing mineral transformation behaviors during Na2CO3-catalyzed
CO2 gasification of high-alumina coal. Energy
Fuels.

[41] Kouzu M., Kajita A., Fujimori A. (2016). Catalytic
activity of calcined scallop
shell for rapeseed oil transesterification to produce biodiesel. Fuel.

[42] Hossain A., Bhattacharyya S. R., Aditya G. (2015). Biosorption of cadmium by waste shell
dust of fresh water mussel lamellidens marginalis: Implications for
metal bioremediation. ACS Sustain. Chem. Eng..

[43] Pal P. P., Bar S., Bera S. K., Sahoo D., Ghorai S. K. (2025). A Multi-Technique
Investigation to Explore the Structural Integrity and Chemical Complexity
of the Brachiopod Lingula anatina (Lamarck, 1801) Shells. J. Struct. Biol..

[44] Gunasekaran S., Anbalagan G., Pandi S. (2006). Raman and infrared spectra of carbonates
of calcite structure. J. Raman Spectrosc..

[45] De
La Pierre M., Carteret C., Maschio L., André E., Orlando R., Dovesi R. (2014). The Raman spectrum of CaCO3 polymorphs
calcite and aragonite: A combined experimental and computational study. J. Chem. Phys..

[46] Gaspard D., Paris C., Loubry P., Luquet G. (2019). Raman investigation
of the pigment families in recent and fossil brachiopod shells. Spectrochim. Acta A Mol. Biomol. Spectrosc..

[47] Arroyo-Loranca R. G., Hernandez-Saavedra N. Y., Hernandez-Adame L., Rivera-Perez C. (2020). Ps19, a novel chitin binding protein
from Pteria sterna
capable to mineralize aragonite plates in vitro. PloS One.

[48] Sampath V., Huang P., Wang F., He D., Zheng Z., Xiao L., Ma C., Li C., Huang L. (2019). Crystalline
organization of nacre and crossed lamellar architecture of seashells
and their influences in mechanical properties. Materialia.

[49] Dufresne W. J., Rufledt C. J., Marshall C. P. (2018). Raman spectroscopy of the eight natural
carbonate minerals of calcite structure. J.
Raman Spectrosc..

[50] Urmos J., Sharma S. K., Mackenzie F. T. (1991). Characterization
of some biogenic
carbonates with Raman spectroscopy. Am. Mineral..

[51] Wehrmeister U., Jacob D., Soldati A., Häger T., Hofmeister W. (2007). Vaterite in freshwater cultured pearls from China and
Japan. J. Gemmol..

[52] Sauer G. R., Zunic W. B., Durig J. R., Wuthier R. E. (1994). Fourier transform
Raman spectroscopy of synthetic and biological calcium phosphates. Calcif. Tissue Int..

[53] Galván I., Jorge A., Solano F., Wakamatsu K. (2013). Vibrational
characterization of pheomelanin and trichochrome F by Raman spectroscopy. Spectrochim. Acta A Mol. Biomol. Spectrosc..

[54] Zakaria F. Z., Mihály J., Sajó I., Katona R., Hajba L., Aziz F. A., Mink J. (2008). FT-Raman and FTIR spectroscopic characterization
of biogenic carbonates from Philippine Venus seashell and Poritessp.
coral. J. Raman Spectrosc..

[55] Pawlukojć A., Leciejewicz J., Ramirez-Cuesta A., Nowicka-Scheibe J. (2015). l-Cysteine:
Neutron spectroscopy, Raman, IR and ab initio study. Spectrochim. Acta A Mol. Biomol. Spectrosc..

[56] Sole C., Drewett N. E., Hardwick L. J. (2014). Insitu
Raman study of lithium-ion
intercalation into microcrystalline graphite. Faraday Discuss..

[57] Bazylewski P., Divigalpitiya R., Fanchini G. (2017). In situ Raman spectroscopy distinguishes
between reversible and irreversible thiol modifications inl-cysteine. RSC Adv..

[58] Dovbeshko G., Fesenko O., Dementjev A., Karpicz R., Fedorov V., Posudievsky O. Y. (2014). Coherent
anti-Stokes Raman scattering enhancement of
thymine adsorbed on graphene oxide. Nanoscale
Res. Lett..

[59] Hu Z., Wang X., Wang W., Zhang Z., Gao H., Mao Y. (2015). Raman spectroscopy
for detecting supported planar lipid bilayers
composed of ganglioside GM1/sphingomyelin/cholesterol in the presence
of amyloid-β. Phys. Chem. Chem. Phys..

[60] Tfaili S., Gobinet C., Josse G., Angiboust J. F., Manfait M., Piot O. (2012). Confocal Raman microspectroscopy
for skin characterization: a comparative study between human skin
and pig skin. Analyst.

[61] Czamara K., Majzner K., Pacia M. Z., Kochan K., Kaczor A., Baranska M. (2015). Raman spectroscopy
of lipids: a review. J. Raman Spectrosc..

[62] Hoang L. H., Hien N. T. M., Chen X. B., Minh N. V., Yang I. S. (2011). Raman spectroscopic
study of various types of tourmalines. J. Raman
Spectrosc..

[63] Marin F. (2012). The formation
and mineralization of mollusk shell. Front.
Biosci..

[64] Sun J., Bhushan B. (2012). Hierarchical structure and mechanical properties of
nacre: a review. RSC Adv..

[65] Dauphin Y., Luquet G., Salome M., Bellot-Gurlet L., Cuif J. (2018). Structure and composition of Unio
pictorum shell: arguments for the
diversity of the nacroprismatic arrangement in molluscs. J. Microsc..

[66] Chakraborty A., Parveen S., Chanda D. K., Aditya G. (2020). An insight into the
structure, composition and hardness of a biological material: the
shell of freshwater mussels. RSC Adv..

[67] Yourdkhani M., Pasini D., Barthelat F. (2011). Multiscale mechanics and optimization
of gastropod shells. J. Bionic Eng..

[68] Bhushan B. (2009). Biomimetics:
lessons from nature–an overview. Philos.
Trans. R. Soc. A.

[69] Espinosa H. D., Rim J. E., Barthelat F., Buehler M. J. (2009). Merger of structure
and material in nacre and bone – Perspectives on de novo biomimetic
materials. Prog. Mater. Sci..

[70] Nudelman F. (2015). Nacre biomineralisation:
A review on the mechanisms of crystal nucleation. Semin. Cell Dev. Biol..

[71] Heinemann F., Launspach M., Gries K., Fritz M. (2011). Gastropod
nacre: Structure,
properties and growth — Biological, chemical and physical basics. Biophys. Chem..

[72] Checa A. G., Rodríguez-Navarro A. B. (2005). Self-organisation
of nacre in the
shells of Pterioida (Bivalvia: Mollusca). Biomaterials.

[73] Barthelat F., Li C. M., Comi C., Espinosa H. D. (2006). Mechanical properties
of nacre constituents and their impact on mechanical performance. J. Mater. Res..

[74] Du F., Alghamdi S., Yang J., Huston D., Tan T. (2023). Interfacial
Mechanical Behavior in Nacre of Red Abalone and Other Shells: A Review. ACS Biomater. Sci. Eng..

[75] Goswami A. (2021). A comparative
study on the mechanical and structural design of nacre in gastropod
and bivalve molluscs. J. Mech. Behav. Biomed.
Mater..

[76] Katti K. S., Katti D. R. (2006). Why is nacre so tough and strong?. Mater. Sci. Eng. C.

[77] Katti K. S., Katti D. R., Pradhan S. M., Bhosle A. (2005). Platelet interlocks
are the key to toughness and strength in nacre. J. Mater. Res..

[78] Marie B., Le Roy N., Zanella-Cléon I., Becchi M., Marin F. (2011). Molecular evolution of mollusc shell
proteins: Insights from proteomic
analysis of the edible mussel Mytilus. J. Mol.
Evol..

[79] Şereflişan H. (2023). Structural
and biological characterization of two freshwater mussel shells (Bivalvia:
Unionidae). Turk. J. Zool..

[80] Zhao Y., Sun X., Zhang G., Trewyn B. G., Slowing I. I., Lin V. S.-Y. (2011). Interaction
of mesoporous silica nanoparticles with human red blood cell membranes:
Size and surface effects. ACS Nano.

[81] Ateş A., Aydemir B., Öksüz K.
E. (2024). Investigation of physicochemical
and biological properties of boron-doped biochar. Biomass Convers. Biorefin..

[82] Gao L., Chen J., Feng W., Song Q., Huo J., Yu L., Liu N., Wang T., Li P., Huang W. (2020). A multifunctional
shape-adaptive and biodegradable hydrogel with hemorrhage control
and broad-spectrum antimicrobial activity for wound healing. Biomater. Sci..

[83] Chen F., Cao X., Yu J., Su H., Wei S., Hong H., Liu C. (2017). Quaternary ammonium
groups modified starch microspheres for instant
hemorrhage control. Colloids Surf. B Biointerfaces..

