# Supplementary material for: Multiscale Characterization and Bioactivity of Freshwater Unionid Mussel Shells as Sustainable Natural Biomaterials
Source: ACS Omega. 2026 Jun 1;11(23):34526–42. doi: 10.1021/acsomega.6c02594 (PMC13280912; doi:10.1021/acsomega.6c02594)

Supporting Information

Multiscale Characterization and Bioactivity of Freshwater Unionid Mussel Shells as Sustainable Natural Biomaterials

Kerim Emre Öksüz[1,2]*, Hülya Şereflişan[3], Erkan Uğurlu[3]

[1]Sivas Cumhuriyet University, Department of Metallurgical and Materials Engineering, Sivas, 58140, Türkiye

[2]Sivas Cumhuriyet University, Institute of Science and Technology, Department of Bioengineering, Sivas, 58140, Türkiye

[3]İskenderun Technical University, Faculty of Marine Sciences and Technology, 31200, İskenderun, Hatay, Türkiye

**Corresponding author:* kerimemreoksuz@gmail.com, emre.oksuz@cumhuriyet.edu.tr

Highlights

- Exploration of natural biomaterials: The study investigates into the unionid freshwater mussel shells of *A. anatina* and *U. delicatus*, presenting a comprehensive examination for the first time.

- Detailed analysis: The experimental research offers a thorough investigation into the unique microstructure, bio-interface, shell morphology, and spectral properties of mussel shells within their ecological system.

- Natural biomaterial qualities: The manuscript highlights the remarkable cytotoxic properties and hem compatibility exhibited by mussel shells, positioning them as promising natural biomaterials.

.

Graphical Abstract

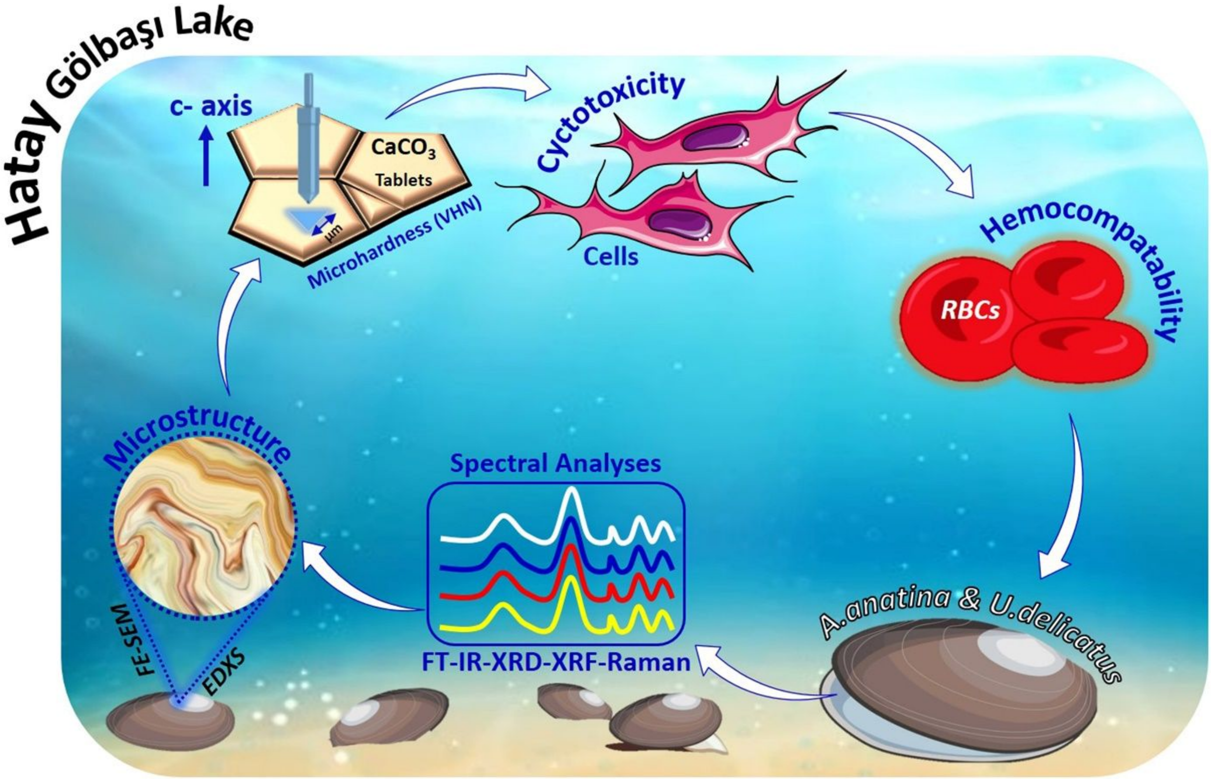

Supplement: Supplementary file 1 [file ao6c02594_si_001.pdf]
